# The formation of chaperone-rich GET bodies depends on the tetratricopeptide repeat region of Sgt2 and is reversed by NADH

**DOI:** 10.1242/jcs.263616

**Published:** 2025-03-20

**Authors:** Jonas Jennrich, Ákos Farkas, Henning Urlaub, Blanche Schwappach, Katherine E. Bohnsack

**Affiliations:** ^1^ Department of Molecular Biology, University Medical Centre Göttingen, Justus-von-Liebig-Weg 11, 37077 Göttingen, Germany; ^2^ Max Planck Institute for Multidisciplinary Sciences, Bioanalytical Mass Spectrometry, Am Faßberg 11, 37077 Göttingen, Germany; ^3^ Institute for Clinical Chemistry, University Medical Centre Göttingen, Robert-Koch-Straße 40, 35075 Göttingen, Germany

**Keywords:** Chaperone, Protein targeting, Get3 ATPase, Membraneless organelle, Glucose metabolism, Cellular stress

## Abstract

The guided-entry of tail-anchored proteins (GET) pathway is a post-translational targeting route to the endoplasmic reticulum (ER). Upon glucose withdrawal, the soluble GET proteins re-localize to dynamic cytosolic foci, here termed GET bodies. Our data reveal that the pre-targeting complex components, Sgt2 and the Get4–Get5 heterodimer, and the Get3 ATPase play important roles in the assembly of these structures in *Saccharomyces cerevisiae*. More specifically, the TPR region of Sgt2 is required as a GET body scaffold. Systematic compositional analyses of GET bodies reveal their chaperone-rich nature and the presence of numerous proteins involved in metabolic processes. Temporal analyses of GET body assembly demonstrate the sequential recruitment of different chaperones, and we discover the requirement of Sis1 and Sti1 for maintaining the dynamic properties of these structures. *In vivo*, NADH derived from the oxidation of ethanol to acetaldehyde can induce GET body disassembly in a reaction depending on the alcohol dehydrogenase Adh2 and *in vitro*, addition of NADH resolves GET bodies. This suggests a mechanistic basis for their formation and disassembly in response to the metabolic shift caused by glucose withdrawal and re-addition.

## Teaser

**Highlighted Article:** Upon glucose deprivation, components of the guided entry of tail-anchored proteins pathway re-localize to dynamic cytosolic foci enriched in metabolic enzymes and chaperones, which are resolved by NADH.

## INTRODUCTION

Membrane protein biogenesis requires not only their translation on cytosolic ribosomes, but also targeting to appropriate membrane destinations. Nascent membrane proteins are inherently aggregation-prone due to the hydrophobic transmembrane segments (TMSs) that allow their integration into lipid bilayers, and impaired targeting can cause their aggregation in the cytosol, leading to reduced membrane functionality and proteotoxic stress. For membrane proteins carrying C-terminal targeting signals, such as tail-anchored (TA) proteins, post-translational targeting is necessary. TA proteins are involved in diverse processes in the endoplasmic reticulum (ER), Golgi, inner nuclear and peroxisomal membranes including regulating vesicular transport, apoptosis, lipid biosynthesis and protein quality control (Borgese et al., 2003). Many TA proteins utilize the guided entry of tail-anchored proteins (GET) pathway [*Saccharomyces cerevisiae* (yeast); known as the transmembrane recognition complex (TRC) pathway in mammals] to reach their target membranes via their initial membrane insertion into the ER (Favaloro et al., 2008; Schuldiner et al., 2008; Stefanovic and Hegde, 2007).

Clients of the GET pathway are captured by a pre-targeting complex composed of the homodimeric heat-shock cognate co-chaperone Sgt2 and the Get4–Get5 heterodimer (Get5 is also known as Mdy2; Hu et al., 2006) (Cho and Shan, 2018; Jonikas et al., 2009; Mariappan et al., 2011; Wang et al., 2010). The pre-targeting complex associates with ribosomes so is poised to capture nascent clients (Zhang et al., 2016, 2021). Their TMSs are shielded by Sgt2, which contains a glutamine- and methionine-rich C-terminal region responsible for substrate binding (Liou and Wang, 2005; Wang et al., 2010). Sgt2 also contains a tetratricopeptide repeat (TPR), proposed to function as a platform for interactions with other chaperones, and its N-terminal domain mediates dimerization and contacts the other pre-targeting complex components (Chartron et al., 2011; Krogan et al., 2006; Krysztofinska et al., 2017; Liou et al., 2007; Wang et al., 2010). The Get4–Get5 heterodimer effects the handover of TA proteins from the pre-targeting complex to the chaperone Get3 (Chartron et al., 2010, 2011), where the TMS of the client protein is shielded within a hydrophobic pocket of the Get3 ATPase. Binding of the TA protein induces ATP hydrolysis by Get3, leading to its dissociation from the pre-targeting complex (Chartron et al., 2010; Gristick et al., 2014; Rome et al., 2013). TA protein- and ADP-bound Get3 then associates with the GET receptor in the ER membrane. Binding of Get3 to the GET receptor, composed of Get1 and Get2, promotes ADP release, triggering conformational changes in Get3 that drive TA protein delivery for insertion into the ER membrane and recycling of Get3 (Kubota et al., 2012; Mariappan et al., 2011; Rome et al., 2014; Schuldiner et al., 2008; Stefer et al., 2011; Wang et al., 2011).

Although TA proteins are the canonical substrates of the GET pathway, the client spectrum of the GET pathway extends beyond TA proteins (Ast et al., 2013; Farkas et al., 2022). Furthermore, considerable redundancy within the targeting network is observed and most TA proteins can still be targeted to the ER when the GET pathway is perturbed (Aviram and Schuldiner, 2017; Chitwood et al., 2018; Guna et al., 2018; Jonikas et al., 2009; Schuldiner et al., 2008). This flexible protein targeting network is essential to meet the changing demands for particular membrane proteins in different conditions. The challenge of adapting to the fluctuating requirements for specific membrane proteins can be overcome by regulated gene expression programs coupled to the utilization of specific targeting pathways (Farkas et al., 2022).

Dynamic adaptation of protein targeting is highly pertinent to yeast, which encounter various circumstances within their natural environments, such as fluctuating temperatures, oxidative stresses and diverse nutrient availabilities. When glucose is available, *S. cerevisiae* utilizes fermentative metabolism despite the presence of oxygen and represses the use of alternative carbon sources as well as gluconeogenesis (De Deken, 1966; De Vit et al., 1997; Rodrigues et al., 2006; Treitel and Carlson, 1995). The cellular response to changing environmental and metabolic conditions is multifaceted (de Nadal et al., 2011; Farkas et al., 2022; Hinnebusch, 1988, 1994; Posas et al., 1998) and, at the post-translational level, an efficient mechanism to dynamically modulate the cellular proteome is the sequestration of specific proteins. Examples of membraneless compartments include stress granules and P-bodies, which can be induced by a variety of conditions including glucose withdrawal, osmotic stress and UV irradiation, Sec-bodies, which form upon amino acid starvation, G-bodies, which assemble in response to hypoxic stress (Jin et al., 2017; Teixeira et al., 2005; Zacharogianni et al., 2014), and Q-bodies (Escusa-Toret et al., 2013; Sathyanarayanan et al., 2020), which form in response to proteotoxic stress, chronological aging or upon inhibition of mTOR signaling. Formation of such entities can be driven by liquid–liquid phase separation (LLPS) in which the high concentration of multivalent interactions between the disordered regions of constituent proteins or RNAs induces the spontaneous transition into two distinct phases (Banani et al., 2016; Protter et al., 2018; Sanders et al., 2020; Yang et al., 2020). Compositional analyses of condensates formed in response to different stress conditions reveal distinct proteomes and, in some cases, transcriptomes (Buchan et al., 2011; Kedersha et al., 2002; Stöhr et al., 2006). For example, stress granules are suggested to serve as sequestration sites for translation initiation components, non-translating mRNAs and many additional mRNA-associated proteins (Buchan et al., 2008; Grousl et al., 2009; Hoyle et al., 2007; Kedersha et al., 1999, 2002; Stöhr et al., 2006). The formation of membraneless compartments is also thought to modulate the properties of the cytoplasm (Xie et al., 2024) to allow for an efficient stress response.

In the context of oxidative stress, Get3 can act as an ATP-independent chaperone holdase (Powis et al., 2013; Ulrich et al., 2022; Voth et al., 2014). Changes in the redox status of conserved cysteine residues in oxidative stress conditions alters Get3 disulfide bridges, modulating nucleotide binding and inducing oligomerization. Protein unfolding is a hallmark of oxidative stress, and Get3 is implicated in facilitating the handover of denatured proteins to ATP-dependent chaperones for refolding, thus contributing to the alleviation of oxidation-induced proteotoxic stress (Ulrich et al., 2022). Furthermore, Get3, which is normally distributed throughout the cytosol and transiently associated with the ER membrane, re-localizes to cytosolic foci in the context of glucose starvation (Powis et al., 2013). Re-addition of glucose triggers rapid dissipation of these foci, suggesting that they are dynamic assemblies, rather than irreversible aggregates. Here, we dissect the requirement of different GET pathway proteins for the formation of stress-induced GET bodies, analyze the necessity of individual regions of Sgt2 for this, systematically investigate the composition of GET bodies, and explore the physiological basis of GET body formation and their dissolution.

## RESULTS

### Dynamic cytosolic foci containing soluble components of the GET pathway form upon acute glucose withdrawal

**Fig. 1.**
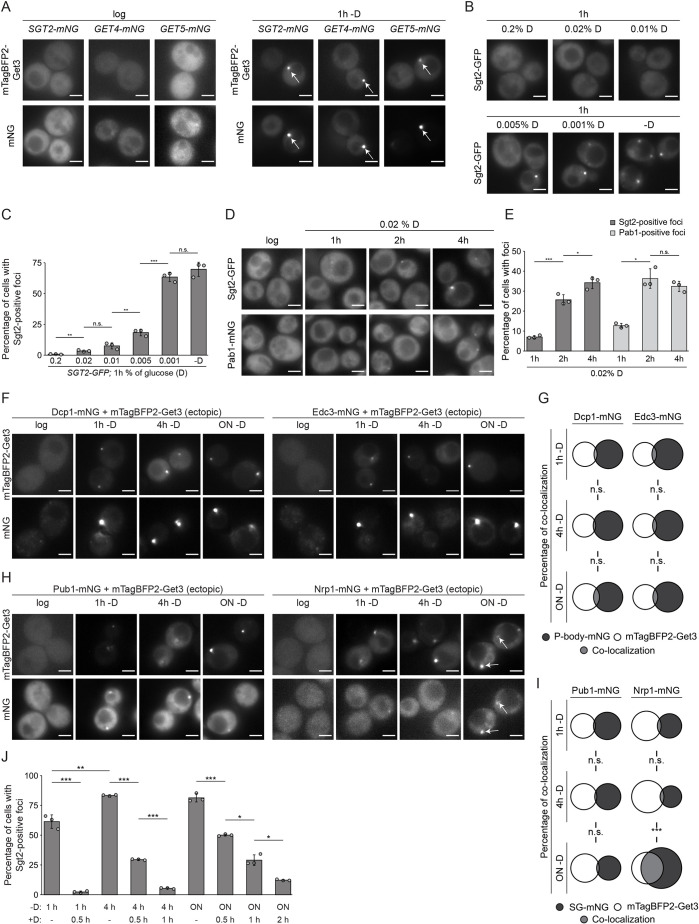
**Acute glucose withdrawal leads to re-localization of soluble GET pathway proteins to cytosolic foci that are distinct from P-bodies and stress granules.** (A) Fluorescence microscopy of cells expressing mNG-tagged GET pathway components and mTagBFP2–Get3 in logarithmic phase (log) or after 1 h in the absence of glucose (−D). Arrows indicate colocalization. (B) Fluorescence microscopy of cells expressing Sgt2–GFP after 1 h in medium containing the indicated amounts of glucose (labeled D). (C) Percentages of cells containing foci from experiments as in B. (D) Fluorescence microscopy of cells expressing Sgt2–GFP or Pab1–mNG in log phase or after the indicated times in medium containing 0.02% D. (E) Percentages of cells containing foci in D. (F–I) Fluorescence microscopy of cells expressing mNG-tagged P-body (F) or stress granule (H) marker proteins and mTagBFP2–Get3. Percentages of colocalization between mTagBFP2–Get3-positive foci and foci containing a P-body (G) or stress granule marker (I). Sizes of circles represent the proportions of the total foci counted. Arrows in H highlight colocalization. (J) Percentages of cells containing Sgt2-positive foci in cells expressing Sgt2–mNG and mTagBFP2–Get3 in log phase or after the indicated times in medium lacking glucose (−D). +D indicates addition of 2% glucose. Scale bars: 2 µm. All panels show results for or representative of *n*=3 biological replicates. Error bars are mean±s.d.; n.s., not significant (*P*>0.05); *P<0.05; **P<0.01; ***P<0.005 (Welch's *t*-test). ON, overnight.

It has been observed that Get3 and Get5 re-localize to cytosolic foci upon glucose withdrawal (Powis et al., 2013; Cohnen et al., 2010). To systematically explore the recruitment of other components of the GET pathway pre-targeting complex to these foci, yeast strains expressing mNeonGreen (mNG)-tagged Sgt2, Get4 or Get5 as well as mTagBFP2–Get3 were imaged during logarithmic growth and after 1 h in medium devoid of glucose or any other carbon source. During logarithmic growth, all proteins showed diffuse cytosolic localization (Fig. 1A, left). After 1 h in medium lacking glucose, Get3 and all three components of the GET pre-targeting complex colocalized in distinct cytosolic foci (Fig. 1A, right). Foci containing soluble GET proteins and TA proteins are also observed when targeting by the GET pathway is disrupted (Jonikas et al., 2009; Schuldiner et al., 2008); however, the glucose starvation-induced foci are distinct as they do not contain the GET pathway TA protein client Sed5 (Fig. S1A).

Other stress-induced assemblies, such as stress granules, are detected when cells are grown in medium containing reduced levels of glucose (e.g. 0.2%; Sathyanarayanan et al., 2020), so to determine whether such conditions also trigger re-localization of GET components to foci, logarithmically growing cells expressing Sgt2–GFP were transferred to medium devoid of glucose or containing low amounts of glucose (standard medium is 2%). Although 0.2% glucose did not induce foci formation by Sgt2–GFP, and provision of between 0.02% and 0.005% glucose led to foci in only 10–20% of cells, in the absence of glucose or when as little as 0.001% glucose was provided, the majority of cells (∼70%) contained Sgt2–GFP-positive foci (Fig. 1B,C). Extended incubation in medium containing 0.02% glucose increased the proportion of cells containing Sgt2-positive foci up to ∼35% after 4 h, while also inducing the formation of stress granules, marked by Pab1–mNG, to a similar extent (Fig. 1D,E). These results imply that the acute withdrawal of glucose is an important trigger for the re-localization of GET components to cytosolic foci.

The finding that foci containing GET components form only rarely in the presence of low levels of glucose is a difference to what is seen with other stress-induced assemblies, implying that they are distinct entities. In line with this notion, Get3–GFP foci formed when glucose is lacking are not affected by the translation inhibitor cycloheximide, which disrupts stress granules (Kedersha et al., 2000; Powis et al., 2013). To consolidate the distinction between stress granules and P-bodies, and the glucose starvation-induced foci containing GET pathway components, yeast strains expressing mNG-tagged versions of well-established marker proteins of P-bodies and stress granules as well as mTagBFP2–Get3 were starved of glucose for different times. Fluorescence microscopy showed almost no colocalization (Table S1) between Get3 and the P-body marker proteins at any of these time points (Fig. 1F,G; Fig. S2A,B). This was also the case for the stress granule marker proteins eIF4GI, Pub1 and Eap1 (Table S1), but after overnight glucose starvation, the stress granule markers Nrp1, Ngr1 and Pbp1 displayed more extensive colocalization (34%, 16% and 8%, respectively) with Get3-containing foci (Fig. 1H,I; Fig. S2C,D). The colocalization of Get3 with some stress granule marker proteins following prolonged glucose starvation is in line with the previous report of colocalization of Get5 with Pab1 upon glucose withdrawal (Cohnen et al., 2010). Both Hsp42-containing stationary phase granules (SPGs), where Get4–Get5 and Sgt2 have previously been detected (Lee et al., 2018), and Q-bodies (Escusa-Toret et al., 2013) require Hsp42 for their formation so the effect of *HSP42* deletion on the re-localization of GET pathway components to cytosolic foci was monitored. Lack of Hsp42 did not influence glucose withdrawal-induced foci formation by Get3, Get4 or Sgt2 (Fig. S1D,E), indicating distinction from both Q-bodies and Hsp42 SPGs. Together, these data suggest that the foci containing soluble GET pathway components, hereafter termed GET bodies, are distinct entities but that after prolonged glucose withdrawal they share some components with stress granules.

As the extent of colocalization of GET bodies and stress granules varied over time, the numbers of cells containing GET bodies after different durations without glucose were monitored, as well as the dynamics of resolving these assemblies upon re-addition of glucose. GET bodies were present in ∼70% of cells 1 h after glucose withdrawal and this proportion increased to ∼85% after prolonged lack of glucose. The foci formed after 1 h without glucose readily resolved upon re-addition of glucose so that <5% of cells contained foci after 30 min (Fig. 1J; Fig. S1F) (Powis et al., 2013). The lag phase in cells re-initiating division after glucose starvation was longer than 30 min, indicating active dissociation of GET bodies. Foci formed during prolonged absence of glucose also resolved upon re-addition of glucose, but more slowly (Fig. 1I; Fig. S1F), implying that the GET bodies formed remain dynamic structures but that their composition and/or nature changes over time.

### Expression levels of GET pathway components influence GET body formation

**Fig. 2.**
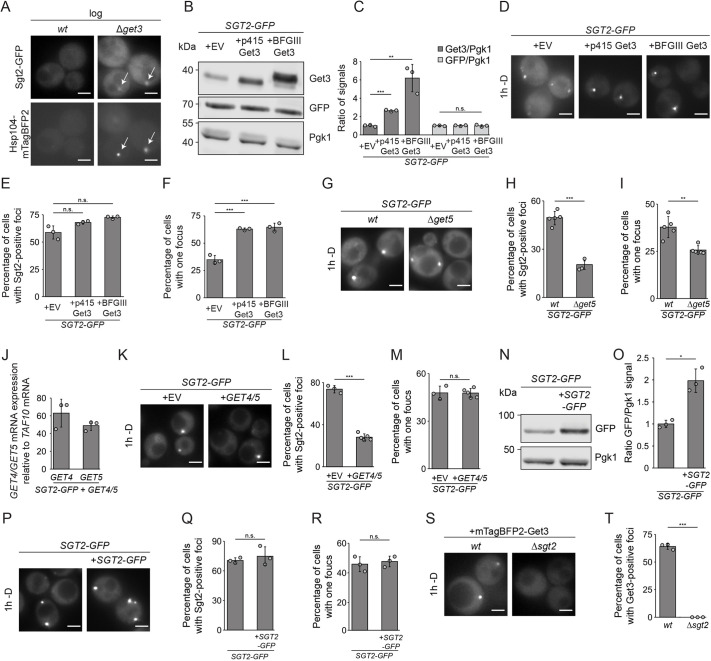
**The absence and overexpression GET proteins influence the proportion of cells with GET bodies and the number of bodies per cell.** (A) Fluorescence microscopy of wild-type (*wt*) or Δ*get3* strains expressing Sgt2–GFP and Hsp104–mTagBFP2 in log phase. Arrows indicate colocalization. (B) Immunoblotting of proteins from cells expressing Sgt2–GFP with an empty vector (EV) or expressing Get3 from the *MET25* (p415 Get3) or *PGK1* (BFGIII Get3) promoters. The shift of Get3 expressed from BFGIII is due to the 3× HA tag. (C) Quantification of Get3 and Sgt2 from experiments as in B, normalized to Pgk1. (D) Fluorescence microscopy of the strains in B in the absence of glucose (−D) for 1 h. (E,F) Percentages of cells in D containing Sgt2–GFP-positive foci (E) and containing only a single focus (F). (G) Fluorescence microscopy of cells expressing Sgt2–GFP in *wt* or Δ*get5* backgrounds after 1 h without glucose. (H,I) Percentages of cells from experiments as in G containing Sgt2–GFP-positive foci (H) and containing only a single focus (I). (J) RT-qPCR of the *GET4* and *GET5* mRNAs relative to the housekeeping gene *TAF10* in *wt* cells. Shown as fold increase relative to wild-type. (K) Fluorescence microscopy of cells expressing Sgt2–GFP in cells transformed with an EV or overexpressing Get4–Get5, after 1 h without glucose. (L,M) Percentages of cells from experiments as in K containing Sgt2–GFP-positive foci (L) and containing only a single focus (M). (N) Immunoblotting of proteins from cells expressing Sgt2–GFP transformed with an EV or expressing Sgt2–GFP from the *MET25* promoter. (O) Sgt2–GFP from experiments as in N was quantified, normalized to Pgk1. (P) Fluorescence microscopy of cells from experiments as in N after 1 h without glucose. (Q,R) Percentages of cells from experiments as in P containing Sgt2–GFP-positive foci (Q) and containing only a single focus (R). (S) Fluorescence microscopy of cells expressing mTagBFP2–Get3 in *wt* or Δ*sgt2* backgrounds after 1 h without glucose. (T) Percentages of cells from experiments as in S containing mTagBFP2-Get3-positive foci. Scale bars: 2 µm. All panels show results for or representative of *n*=3 biological replicates. Error bars are mean±s.d.; n.s., not significant (*P*>0.05); *P<0.05; **P<0.01; ***P<0.005 (Welch's *t*-test).

To explore how the GET pathway components contribute to formation of cytosolic foci, GET bodies were monitored when the levels of GET pathway components were altered by gene deletion or protein overexpression. *GET3* deletion prevents delivery of TA proteins to the GET receptor leading to their aggregation in cytosolic foci during exponential growth (Fig. 2A) (Powis et al., 2013), precluding unambiguous analysis of glucose starvation-induced foci. Ectopic expression of Get3 from different promoters led to mild and strong overexpression of Get3 without influencing Sgt2–GFP expression (Fig. 2B,C). The proportion of cells containing GET bodies was not significantly increased upon Get3 overexpression (Fig. 2D,E); however, the proportion of cells containing only one Sgt2-positive focus was strongly increased (Fig. 2D,F). This suggests that Get3 contributes to the amalgamation of individual foci into larger assemblies, a process observed for other stress-induced assemblies (Decker et al., 2007; Protter and Parker, 2016; Van Treeck and Parker, 2018; Wheeler et al., 2016).

Get4 and Get5 exist as a heterodimer and lack of one protein causes destabilization of the other (Chartron et al., 2010; Liou et al., 2007; Wang et al., 2010). Lack of Get4–Get5 reduced the number of Sgt2-containing foci, and decreased the proportion of cells containing only one focus compared to those containing multiple foci (Fig. 2G–I). Consistent with this, the foci were smaller and less bright in the absence of Get5. Strong co-overexpression of Get4–Get5 (Fig. 2J) led to reduced numbers of cells containing Sgt2–GFP-positive foci but the proportion of cells containing one or multiple foci were not affected (Fig. 2K–M).

Ectopic expression of Sgt2–GFP led to a two-fold increase in the cellular level of Sgt2 (Fig. 2N,O). However, this change, which is similar in extent to the mild overexpression of Get3 (Fig. 2B,C), did not affect the proportion of cells with foci or the ratio of cells containing one or multiple foci (Fig. 2P–R). Strikingly, *SGT2* deletion abolished the association of Get3 with GET bodies (Fig. 2S,T), indicating that Sgt2 is a key component of GET bodies. This is in contrast to the result of Powis et al. (2013) who, upon glucose withdrawal, observed Get3–GFP-positive foci in a Δ*sgt2* strain. Revisiting their Δ*sgt2* strain revealed the presence of a *SGT2* wild-type allele (Fig. S3A,B).

Taken together, these data highlight important roles for the Get4–Get5 heterodimer and Get3 in determining the number of GET bodies per cell, and the requirement of Sgt2 for recruitment of other GET pathway components to these structures.

### Sgt2 acts as a scaffold within the cytosolic foci via its TPR region

**Fig. 3.**
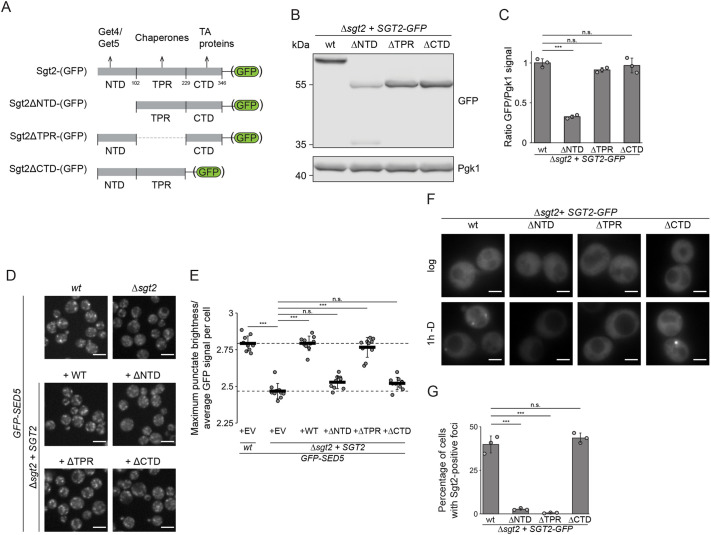
**Sgt2 acts as a scaffold within GET bodies via its TPR.** (A) Schematic representation of Sgt2 with key interaction partners of each region indicated above. Numbers indicate amino acids considered as region borders. (B) Immunoblot of proteins from Δ*sgt2* expressing the GFP-tagged variants as in A from the *MET25* promoter. (C) Quantification of the levels of Sgt2–GFP and truncated versions as in B, normalized to Pgk1. (D) Fluorescence microscopy of a strain expressing GFP–Sed5 in wt (Δ*met15*); Δ*sgt2* was complemented with the indicated versions of Sgt2. (E) Quantification of D. Upper and lower dashed lines represent the ratio between maximum punctate brightness and the mean GFP signal per cell in the wt strain and the Δsgt2 strain with the empty vector control, respectively. (F) Fluorescence microscopy of Δ*sgt2* expressing full-length Sgt2–GFP (wt) or the truncation variants in log phase or after 1 h without glucose (−D). (G) Quantification of the percentages of cells with foci from experiments as in F. Scale bars: 2 µm. Results are for or representative of *n*=3 (B,C,F,G) or *n*=10 (D,E) biological replicates. Error bars are mean±s.d.; n.s., not significant (*P*>0.05); ***P<0.005 (Welch's *t*-test).

We next investigated the contributions of different regions of Sgt2 (Fig. 3A) to its functions in GET body formation and TA protein targeting. In a Δ*sgt2* background, GFP-tagged full-length Sgt2 or truncated variants lacking the TPR (ΔTPR) or C-terminal region (ΔCTD) were expressed similarly (Fig. 3B,C). Expression of N-terminally truncated Sgt2 (ΔNTD) was lower than the ectopically expressed full-length protein (Fig. 3B,C), but on the same level as Sgt2–GFP expressed from the endogenous locus (Fig. S3C,D). No growth defects in logarithmic phase or during the recovery from 1 h deprivation from glucose were observed in cells lacking Sgt2 or upon complementation with either wild-type or truncated Sgt2 (Fig. S3E).

To test the functionality of this complementation system and verify the roles of the Sgt2 regions within the GET pathway, targeting of the TA protein GFP–Sed5, which relies on the GET pathway to reach the Golgi via the ER, was first analyzed in wild-type cells and strains lacking Sgt2, Get4 or Get5 (Jonikas et al., 2009; McDowell et al., 2020). In wild-type, GFP–Sed5 showed a punctate distribution, consistent with its predominant localization in the Golgi (Fig. S3F). To quantify the proportion of GFP–Sed5 in the Golgi system, the ratio between maximum punctate brightness and the average GFP signal per cell was calculated (Fig. S3G). In contrast to what is seen in wild-type cells, in Δ*sgt2*, Δ*get4* and Δ*get5* cells, GFP–Sed5 was detectable in the cytosol and the maximum brightness to average GFP signal ratio was significantly reduced (Fig. S3F,G). Expression of full-length Sgt2 fully restored the Golgi localization of GFP–Sed5 in the Δ*sgt2* strain, whereas expression of Sgt2 lacking either the N- or C-terminus failed to restore targeting of GFP–Sed5, consistent with the roles of these regions in effecting interactions with GET pathway components and clients, respectively (Fig. 3D,E). Strikingly, the TPR region, whose role in TA protein targeting has remained ambiguous (Cho and Shan, 2018; Kohl et al., 2011), was not required for correct localization of GFP–Sed5 (Fig. 3D,E), implying that it is not essential for targeting of canonical TA protein clients by the GET pathway in cells.

We then addressed which regions of Sgt2 are required for its re-localization to cytosolic foci during glucose starvation. Mirroring full-length Sgt2–GFP, Sgt2ΔCTD–GFP was present in the cytosol during exponential growth and formed foci colocalizing with Get3 after 1 h without glucose (Fig. 3F,G; Fig. S3H,I). However, Sgt2ΔNTD–GFP and Sgt2ΔTPR–GFP remained diffusely cytosolic after 1 h without glucose (Fig. 3F,G; Fig. S3H,I), and Get3 was not recruited to foci (Fig. S3H,I), highlighting these regions of Sgt2 as necessary for GET body formation. In the case of the N-terminal domain, this might reflect the importance of this region for Sgt2 dimerization or its ability to bind Get4–Get5, which can bridge interactions to Get3. The discovery that the TPR of Sgt2 is dispensable for TA protein targeting, but necessary for GET body formation, allows dissection of the pre-requisites for, and consequences of, the two functions.

### GET bodies are chaperone-rich and contain proteins involved in metabolism

**Fig. 4.**
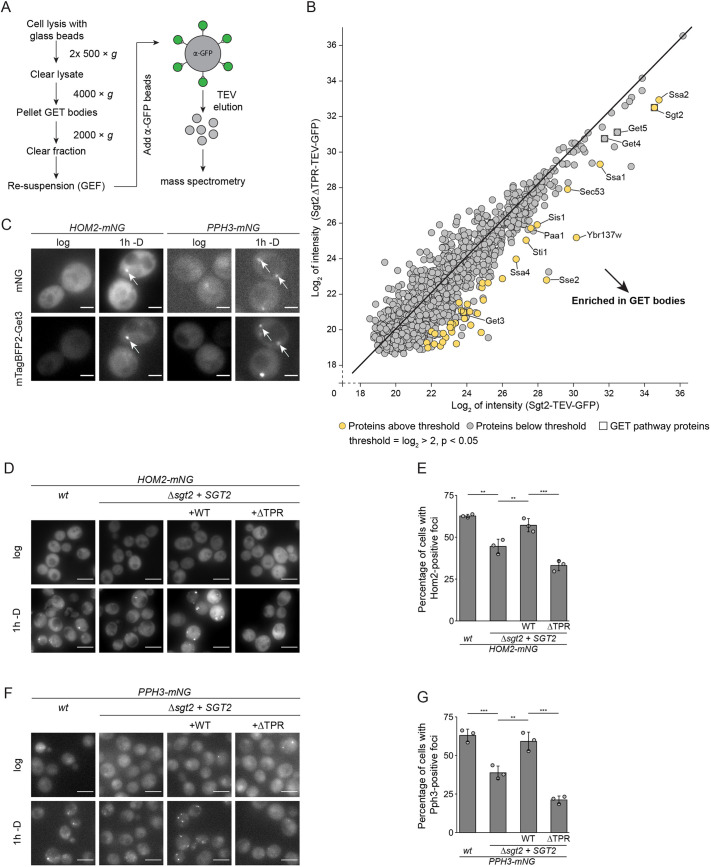
**GET bodies are enriched for chaperones and colocalize with numerous additional proteins.** (A) Workflow for the enrichment of GET bodies. Δ*sgt2* cells expressing Sgt2-TEV–GFP or Sgt2ΔTPR-TEV–GFP were glucose starved for 4 h, lysed and GET bodies enriched by differential centrifugation (GET body-enriched fraction; GEF). Bodies were captured on anti-GFP (α-GFP) beads, specifically eluted and eluates were subjected to mass spectrometry. (B) Average log_2_ intensities of proteins identified in the Sgt2-TEV–GFP and Sgt2ΔTPR-TEV–GFP eluates. Proteins >4-fold enriched significantly (*P*<0.05) are in yellow and GET pathway proteins are boxed. Get5 was >3.3-fold enriched with Sgt2ΔTPR-TEV–GFP compared to Sgt2-TEV–GFP (*P*=0.009), Get4 was >2.6-fold enriched with Sgt2ΔTPR-TEV–GFP compared to Sgt2-TEV–GFP (*P*=0.058). (C) Fluorescence microscopy of yeast expressing Hom2–mNG or Pph3–mNG and ectopically mTagBFP2–Get3 in log phase and after 1 h without glucose (−D). Arrows highlight colocalization. Scale bars: 2 µm. (D–G) Fluorescence microscopy of *wt* or Δ*sgt2* strains expressing Hom2–mNG (D) or Pph3–mNG (F) and, when indicated, full-length Sgt2 (WT) or Sgt2ΔTPR in log phase or after 1 h glucose starvation. Scale bars: 5 µm. Percentages of cells from experiments as in D and F with foci are shown in E and G, respectively. Results are for or representative of *n*=3 biological replicates. Error bars are mean±s.d.; **P<0.01; ***P<0.005 (Welch's *t*-test).

To explore whether proteins other than GET pathway components are present in GET bodies, two complementary approaches were applied to probe their composition – enrichment followed by mass spectrometry (MS) and high-throughput microscopy-based colocalization screening. Enrichment of GET bodies from cells involved differential centrifugation of cell extracts to separate GET bodies and other membraneless organelles displaying similar sedimentation behavior from free proteins and small complexes, followed by immunoprecipitation of GET bodies via Sgt2–GFP, before specific elution by protease-mediated cleavage (Fig. 4A; Fig. S4A). Sgt2ΔTPR–GFP represented an ideal control as this strain is unable to form GET bodies, thus allowing the specific identification of proteins colocalizing with Sgt2 in glucose starvation-induced foci. Analysis of the immunoprecipitation eluates obtained from cells expressing Sgt2-TEV–GFP and Sgt2ΔTPR-TEV–GFP after 4 h without glucose revealed an inventory of proteins specifically enriched with Sgt2-TEV–GFP (Fig. 4B; Table S2). As anticipated, the soluble GET pathway components, Get4, Get5 and Get3 were strongly enriched with Sgt2-TEV–GFP, as was the poorly characterized protein Ybr137w, which has been implicated in TA protein capture in the context of the GET pathway (Wang et al., 2010). Proteins with high abundance that were significantly enriched with GET bodies include the Hsp70 family chaperones Ssa1, Ssa2, Ssa4 and Sse2. Interactions between Hsp70-like chaperones and GET pathway pre-targeting complex components have been observed previously (Wang et al., 2010; Arhzaouy and Ramezani-Rad, 2012), potentially rationalizing the enrichment of these proteins in glucose starvation-induced GET bodies. The inventory of proteins enriched with GET bodies also includes the type II Hsp40-like Hsp70 co-chaperone Sis1 and the Hsp90-like Hsp70 co-chaperone Sti1, as well as the phosphomannomutase Sec53 and the polyamine acetyltransferase Paa1. This suggests that, alongside the GET pathway components, numerous chaperones stably associate with GET bodies.

A proteome-wide microscopy-based colocalization screen was then performed to determine and confirm the association of these proteins with GET bodies and identify additional GET body components not sufficiently stably associated to be co-purified from cells. A library of strains expressing mNG-tagged proteins from their endogenous promoters (Meurer et al., 2018) was transformed with a plasmid for the expression of mTagBFP2–Get3. As the efficient exchange of glucose-containing medium with glucose-free medium on a high-throughput scale is challenging, cells were diluted in medium lacking glucose and supplemented with the non-metabolizable glucose analogue 2-deoxyglucose (2-DG) to mimic conditions of acute glucose withdrawal (Pajak et al., 2019) and induce GET body formation. Consistent with the GET body enrichment approach (Fig. 4B), clear colocalization of Get3, Get4 and Get5 with the with mTagBFP2–Get3 marker was observed, as well as the Ssa1 and Sse2 Hsp70-family chaperones and the Sis1 and Sti1 co-chaperones (Fig. S4B). Ssa2 and Ssa4 were also occasionally found in Get3-positive foci, but this was not the case for Ybr137w, Sec53 or Paa1 (Fig. S4C). As the glucose starvation conditions used for the colocalization screen were not identical to those used for the GET body enrichment approach, the strains expressing mNG-tagged Paa1, Sec53 or Ybr137w, and mTagBFP2–Get3 were starved of glucose for 1 h, 4 h or overnight before visualization. After 1 h without glucose, neither Paa1 nor Sec53 displayed obvious colocalization with Get3-positive foci, but after 4 h and overnight glucose deprivation, colocalization was observed (Fig. S4E), indicating that these proteins are indeed recruited to GET bodies, but only after extended glucose withdrawal. Tagging of Ybr137w caused accumulation in foci during logarithmic growth (Fig. S4D), excluding it from microscopy-based analyses. However, the presence of endogenous Ybr137w in GET bodies enriched from cells suggests that it is likely a GET body component.

The colocalization screen revealed 59 additional proteins representing potential GET body-associated components (Table S3). Various proteins involved in metabolic regulation were identified, such as Hom2, a dehydrogenase required for amino acid biosynthesis, and Pph3, a phosphatase involved in the regulation of nitrogen catabolism. We investigated whether these proteins colocalize with GET bodies under bona fide glucose withdrawal conditions, and whether this depends on the presence of Sgt2 and its TPR. During logarithmic growth, Hom2–mNG and Pph3–mNG were diffusely cytosolic but after 1 h without glucose, they were detectable in cytosolic foci containing mTagBFP2–Get3 (Fig. 4C). In addition to the prominent Pph3 foci colocalizing with Get3, smaller Pph3-positive foci were also observed. In the Δ*sgt2* strain, the numbers of Hom2- and Pph3-positive foci were strongly decreased compared to wild type (Fig. 4D–G). Re-expression of full-length Sgt2 restored the number of foci to be similar to wild type, whereas expression of Sgt2ΔTPR phenocopied the Δ*sgt2* strain, confirming these proteins as bona fide GET body components that depend on the Sgt2 TPR for association.

### Several Hsp70-like chaperones are recruited to GET bodies downstream of Sgt2 and require its TPR region for association

**Fig. 5.**
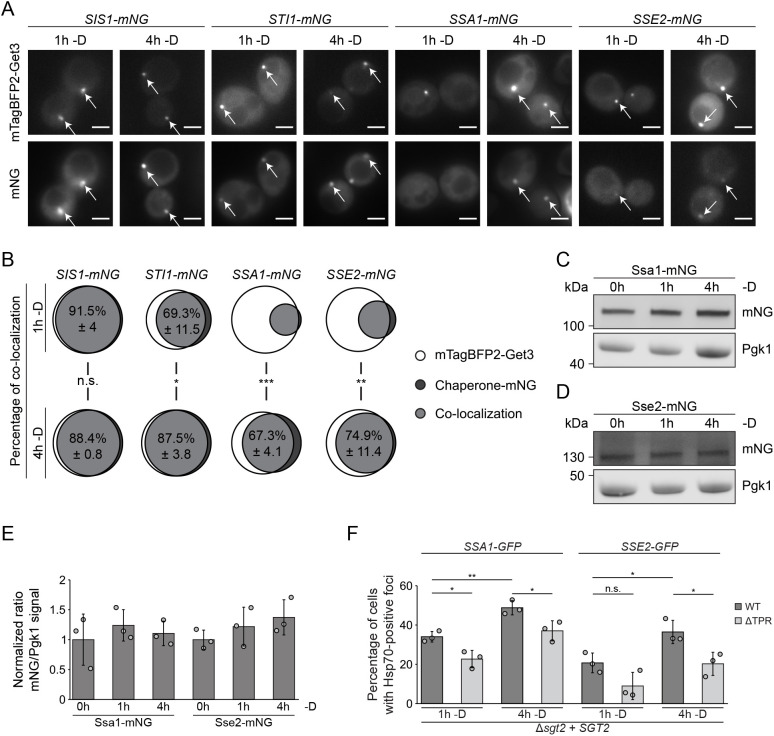
**Hierarchical recruitment of chaperones or co-chaperones to GET bodies.** (A) Fluorescence microscopy of strains expressing mNG-tagged proteins and mTagBFP2–Get3 after 1 h and 4 h glucose starvation (−D). Arrows indicate colocalization. Scale bars: 2 µm. (B) Percentage of colocalization between mTagBFP2–Get3-positive foci and foci positive for an mNG-tagged chaperone. Sizes of circles represent the proportion of the total foci counted. (C,D) Immunoblots of proteins from *wt* yeast expressing Ssa1–mNG (C) or Sse2–mNG (D). (E) Quantification of Ssa1–mNG and Sse2–mNG (from experiments as in C and D, respectively), normalized to Pgk1. (F) The percentages of cells with foci in Δ*sgt2* expressing Ssa1–GFP or Sse2–GFP and wt Sgt2 or Sgt2ΔTPR after 1 and 4 h glucose starvation. Results are for or representative of *n*=3 biological replicates. Error bars are mean±s.d.; n.s., not significant (*P*>0.05); *P<0.05; **P<0.01 (Welch's *t*-test).

Our data suggest hierarchical recruitment of (co-)chaperones to GET bodies, so the colocalization of the identified (co-)chaperones with Get3-containing foci was systematically monitored at different time points following glucose withdrawal. Consistent with the high-throughput microscopy results, at 1 h after glucose withdrawal, only Sis1 and Sti1 showed substantial colocalization with Get3 (Fig. 5A,B). At this time, only a small proportion of Get3-containing foci colocalized with Ssa1 and Sse2, but this represented the majority of Ssa1- and Sse2-containing foci present in cells (Fig. 5B). As anticipated based on the GET body enrichment, all the afore-mentioned (co-)chaperones strongly colocalized with Get3 after 4 h without glucose (Fig. 5A,B; Fig. S5A,B). To determine whether the increased colocalization of Get3 and the Hsp70 family chaperones after 4 h reflects increased expression, their cellular levels were analyzed by immunoblotting. No significant changes were detected (Fig. 5C–E; Fig. S5C–E), implying that the increasing colocalization with Get3 over time reflects their progressive recruitment to GET bodies.

We therefore explored how recruitment of the Hsp70-like chaperones to GET bodies correlates with the presence of the TPR region of Sgt2. When Sgt2ΔTPR was expressed, the number of Ssa1-containing foci was significantly reduced after 1 and 4 h without glucose (Fig. 5F, Fig. S5F), whereas for Sse2, the number of foci was reduced after 1 h, but this only became significant after 4 h glucose deprivation (Fig. 5F; Fig. S5F). These results confirm Ssa1, Ssa2, Ssa4 and Sse2 as GET body components and demonstrate that recruitment of Hsp70-like chaperones to these assemblies is strongly enhanced by the Sgt2 TPR region.

### Sis1 and Sti1 contribute to initiating GET body formation and are required for its disassembly

**Fig. 6.**
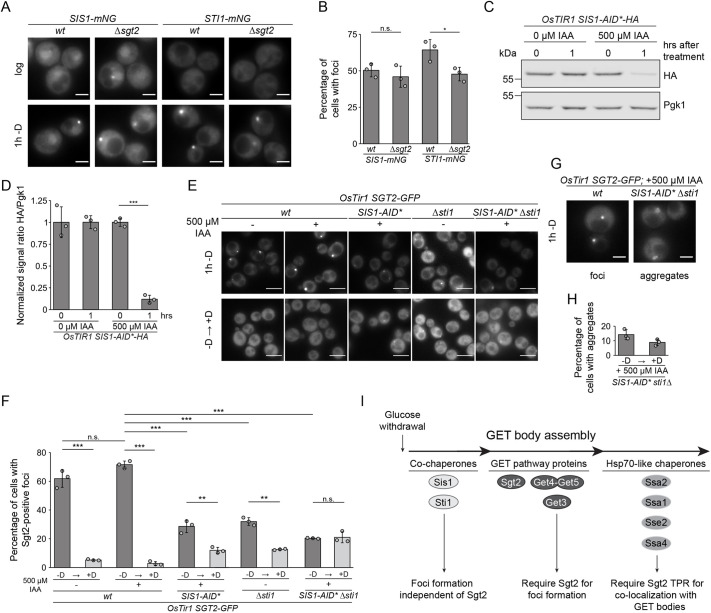
**The co-chaperones Sis1 and Sti1 contribute to initiating GET body formation and are required for disassembly upon re-addition of glucose.** (A) Fluorescence microscopy of strains expressing Sis1–mNG or Sti1–mNG in *wt* or Δ*sgt2* backgrounds in log phase or after 1 h glucose starvation (−D). Scale bars: 2 µm. (B) Percentages of cells from experiments as in A with foci. (C) Immunoblot of proteins from a strain expressing *Os*Tir1 and Sis1-AID*–HA treated with and without 500 µM IAA for the indicated times. (D) Sis1-AID*–HA levels from experiments as in C were quantified and normalized to Pgk1. (E) Fluorescence microscopy of strains expressing *Os*Tir1 and Sgt2–GFP together with Sis1-AID*–HA, Δ*sti1* or both. + indicates IAA treatment prior to glucose starvation. Images in upper row acquired after 1 h glucose starvation, in lower row 30 min after glucose re-addition. Scale bars: 5 µm. (F) Percentage of cells with foci from experiments as in E. (G,H) Fluorescence microscopy of cells expressing Sgt2–GFP in *wt* or SIS1-AID* Δ*sti1* after IAA treatment and 1 h glucose starvation. Images show representative examples of cells with foci and with aggregates in G, and percentages of cells with aggregates in the strain lacking *sti1* in H. Scale bars: 2 µm. (I) Scheme of chaperone recruitment to GET bodies. Results are for or representative of *n*=3 biological replicates. Error bars are mean±s.d.; n.s., not significant (*P*>0.05); *P<0.05; **P<0.01; ***P<0.005 (Welch's *t*-test).

The finding that Sis1 and Sti1 strongly colocalize with Sgt2 in cytosolic foci after 1 h glucose starvation (Fig. 5A,B) raised the question of whether their recruitment to foci depends on Sgt2. The proportion of cells with Sis1–mNG-containing foci was unaffected by lack of Sgt2 and the number of cells with Sti1–mNG-containing foci was only mildly reduced in Δ*sgt2* (Fig. 6A,B), indicating that Sgt2 is largely dispensable for their assembly into cytosolic granules. Nucleation of Sis1 and Sti1 therefore likely precedes the recruitment of Sgt2 and the other GET components. The effect of lack of Sis1 and Sti1 on the ability of Sgt2 to form glucose starvation-induced foci was therefore monitored. As *SIS1* is essential, Sis1–HA was expressed with an auxin-inducible degron (AID) tag in a strain overexpressing *Oryza sativa* TIR1 to allow acute depletion of Sis1 upon addition of auxin (Nishimura et al., 2009) (Fig. 6C,D). Treatment of wild-type cells with auxin did not significantly affect the proportion of cells containing GET bodies; however, depletion of Sis1 or deletion of *STI1* led to substantial decreases in the numbers of cells with Sgt2-containing foci (Fig. 6E, upper row; Fig. 6F, dark gray bars). The reduction in GET bodies was further exacerbated by co-depletion or deletion of Sis1 and *STI1* (Fig. 6E, upper row; Fig. 6F, dark gray bars), suggesting that these proteins function cooperatively to promote the initial steps of GET body formation.

As Sis1 has previously been implicated in promoting the clearance of stress granules (Walters et al., 2015), the requirement of Sis1 and Sti1 for the resolution of GET bodies upon glucose re-addition was explored. Following glucose deprivation, supplementing wild-type yeast with glucose led to almost complete dissolution of foci (<5%) within 1 h, irrespective of prior treatment of the cells with auxin (Fig. 6E, lower row; Fig. 6F, light gray bars). By contrast, when Sis1 was depleted or *STI1* was deleted, the proportion of cells with Sgt2-containing foci 1 h after re-addition of glucose was almost twofold higher (Fig. 6E, lower row; Fig. 6F, light gray bars). When depletion of Sis1 was combined with deletion of *STI1*, ∼25% of cells still contained GET bodies 1 h after re-addition of glucose. Owing to the low number of GET bodies formed in the absence of these co-chaperones, re-addition of glucose did not significantly alter the proportion of cells containing GET bodies (Fig. 6E, lower row; Fig. 6F, light gray bar). In cells lacking both Sis1 and Sti1, Sgt2–GFP was not only detected in the bright, spherical foci characteristic of GET bodies, but was also detected in other heterogeneous structures both after 1 h glucose starvation and also following re-addition of glucose (Fig. 6G,H). These results suggest a hierarchical assembly path of GET bodies in which the co-chaperones Sis1 and Sti1 are nucleating factors for the assembly of GET pathway components, which in turn, allow the recruitment of Hsp70-like chaperones (Fig. 6I).

### GET body dynamics are influenced by the cellular NADH level

**Fig. 7.**
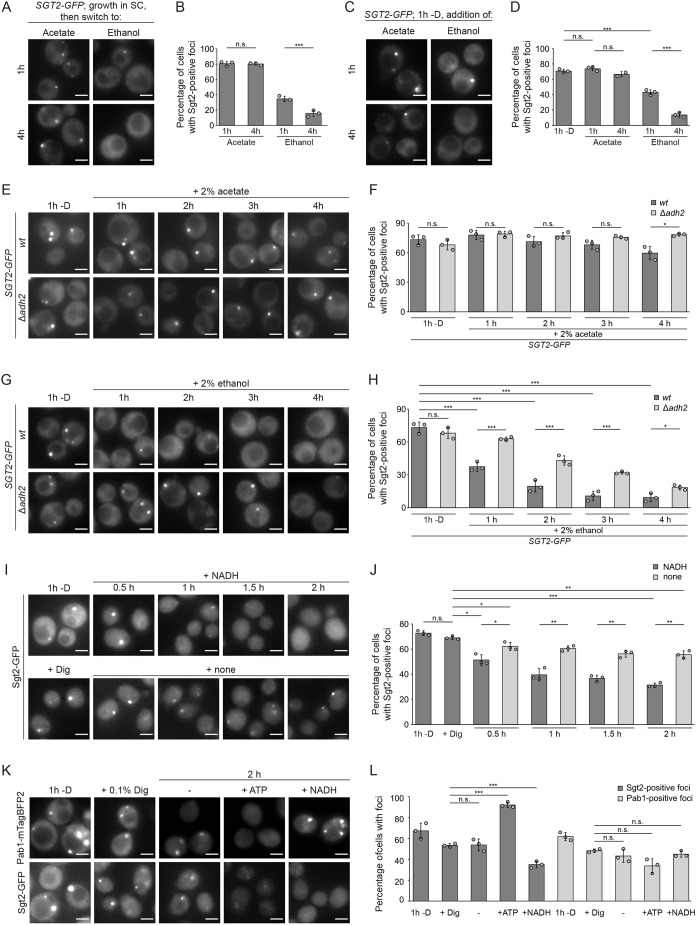
**GET bodies formed upon glucose starvation are resolved by addition of NADH.** (A) Fluorescence microscopy of a strain expressing Sgt2–GFP grown in SC medium and then transferred into medium with 2% (w/v) acetate and 2% (v/v) ethanol as carbon sources 1 and 4 h after the switch. (B) Percentages of cells with foci from experiments as in A. (C) Fluorescence microscopy of a strain expressing Sgt2–GFP after 1 h glucose starvation (−D) and after re-addition of the indicated carbon sources for 1 and 4 h. (D) Percentages of cells with foci from experiments as in C. (E–H) Cultures of *wt* and Δ*adh2* strains expressing Sgt2–GFP starved of glucose for 1 h were divided in two, and medium containing either 2% acetate (E) or 2% ethanol (G) was added. Fluorescence microscopy images were taken after the indicated times. Note that images taken at 1 h (−D) were obtained from the same cultures. Percentages of cells with foci from experiments as in E and G are shown in F and H, respectively. (I) Fluorescence microscopy of a strain expressing Sgt2–GFP after 1 h glucose starvation and treatment with 0.1% digitonin (Dig). Images were taken at the indicated time points after NADH addition. (J) Percentages of cells with foci from experiments as in I. (K) Fluorescence microscopy of a strain expressing Pab1–mTagBFP2 and Sgt2–GFP after 1 h glucose starvation and digitonin treatment. ATP, NADH or neither (−) were added and images taken after 2 h. (L) Percentages of cells with Pab1–mTagBFP2 or Sgt2–GFP foci from experiments as in K. Scale bars: 2 µm. Results are for or representative of *n*=3 biological replicates. Error bars are mean±s.d.; n.s., not significant (*P*>0.05); *P<0.05; **P<0.01; ***P<0.005 (Welch's *t*-test).

*S. cerevisiae* derives energy via aerobic fermentation when glucose is abundant. To gain insight into intracellular cues influencing GET body dynamics, yeast cells expressing Sgt2–GFP were switched into medium lacking glucose, or containing acetate or ethanol, and the numbers of Sgt2–GFP-containing foci were monitored. Similar to cells switched to medium lacking any carbon source (e.g. Fig. 1B), cells in medium containing acetate presented Sgt2–GFP foci in ∼80% of cells (Fig. 7A,B). By contrast, when ethanol was present, less than 40% of cells contained GET bodies after 1 h, and this reduced to ∼20% after 4 h (Fig. 7A,B), implying that acetate mimics glucose withdrawal, but the provision of ethanol bypasses the trigger that induces re-localization of the GET pathway components. Consistent with this, when yeast deprived of a carbon source were transferred to medium containing acetate, the proportion of cells containing GET bodies was maintained (Fig. 7C,D), but upon addition of ethanol-containing medium, the foci progressively resolved so that after 4 h <20% of cells displayed GET bodies (Fig. 7C,D).

When glucose is abundant, it is mainly directed towards glycolysis and pyruvate is slowly channeled into the tricarboxylic acid cycle to drive energy production via the electron transport chain. However, fermenting yeast display low levels of mitochondrial function (Chen and Clark-Walker, 2000), forcing cells to use other reactions to recycle the NAD^+^ required for ATP production from glycolysis. Ethanol formation promotes NADH consumption under these conditions because ethanol can leave cells (Kotyk and Alonso, 1985), enabling continued oxidation of NADH to NAD^+^. When glucose is limiting, yeast switch from rapid fermentative growth to slower growth relying on aerobic respiration. If ethanol is supplied as a carbon source, it is converted into acetaldehyde by the alcohol dehydrogenase Adh2, concomitant with the reduction of NAD^+^ to NADH. In an additional oxidation reaction conferring electrons to NAD^+^ to produce NADH, acetaldehyde is further converted into acetate that then requires ATP-dependent activation to enter the metabolic flux. As the presence of ethanol, but not acetate, resolved GET bodies (Fig. 7C,D), the effect of deleting *ADH2* on the resolution of Sgt2–GFP foci in the presence of ethanol and acetate was investigated. GET bodies were not resolved by addition of acetate, irrespective of whether *ADH2* was expressed or not (Fig. 7E,F). However, whereas in wild-type cells, GET bodies were progressively resolved when ethanol was supplied, in Δ*adh2* cells, the proportion of cells containing GET bodies reduced, but more slowly (Fig. 7G,H), suggesting that the Adh2-catalyzed reaction is relevant for the dynamics of GET bodies.

As lack of Adh2 impairs GET body resolution, it is possible that elevated levels of intracellular NADH might be a trigger inducing disassembly of these structures. To explore this *in vitro*, we semi-permeabilized cells expressing Sgt2–GFP grown in the absence of a carbon source for 1 h with digitonin (Berłowska et al., 2006) and then supplied NADH. Although cells are not viable after semi-permeabilization, this allowed a comparative analysis of GET body dynamics in different conditions. Semi-permeabilization initially led to a mild decrease in the proportion of cells containing GET bodies, but the proportion of cells containing GET bodies was then barely affected for 2 h (Fig. 7I,J). However, upon addition of NADH to the semi-permeabilized cells, the proportion of cells containing Sgt2-positive foci was gradually reduced by more than half over the course of 2 h (Fig. 7I,J), suggesting that GET bodies disassemble in the presence of NADH. Exogenous addition of ATP resolves stress granules (Sathyanarayanan et al., 2020), so to explore the specificity of the effect of NADH on GET bodies, glucose-starved cells expressing either Sgt2–GFP or Pab1–mTagBFP2 were semi-permeabilized, then treated with ATP or NADH. The proportion of cells with stress granules was mildly reduced by ATP, but it was unaffected by NADH (Fig. 7K,L). By contrast, addition of NADH significantly decreased the proportion of cells containing Sgt2-positive foci whereas treatment with ATP did not (Fig. 7K,L). These data suggest that elevated NADH levels specifically promote the disassembly of GET bodies.

## DISCUSSION

A key cellular response to encountering physical or environmental challenges is the formation of membraneless organelles. Here, we characterize a membraneless organelle formed in response to acute glucose withdrawal that is distinguished by the presence of components of the GET targeting pathway. Get3 re-localizes to cytosolic foci in the absence of glucose (Powis et al., 2013), and the GET pathway pre-targeting complex components also colocalize with these foci (Fig. 1). Based on systematic compositional analyses, and by comparison with a panel of well-established marker proteins of P-bodies and stress granules, we reveal GET bodies as distinct entities within the complement of membraneless compartments in yeast (Fig. 1; Fig. S2; Tables S2 and S3). The finding that, after prolonged glucose starvation, there is limited colocalization of stress granule proteins with GET bodies could (1) reflect a physical propensity of stress granule or GET body proteins to non-specifically merge with established membraneless organelles, (2) indicate the exchange of components between GET bodies and stress granules as has been suggested for other membraneless organelles (Buchan et al., 2008; Kedersha et al., 2005) or (3) suggest that extended absence of glucose triggers additional stresses that drive the recruitment of these stress-sensitive proteins to GET bodies.

Consistent with a role of the GET proteins in body formation, alterations in their levels influence the GET body phenotype (Fig. 2). Although wild-type cells often display several GET bodies per cell, overexpression of Get3 increases the proportion of cells with only one GET body, and lack of Get4–Get5 leads to numerous smaller foci per cell, suggesting functions in the coalescence of individual GET bodies into single entities. This draws parallels with stress granules that undergo extensive remodeling and ATP-dependent fusion (Jain et al., 2016). An important feature of the soluble GET pathway proteins is their ability to form multivalent interactions; Sgt2 is a dimer that physically interacts with the Get4–Get5 complex, which itself dimerizes and also interacts with the Get3 dimer (Farkas and Bohnsack, 2021). Consistent with this, recruitment of Get3 to foci upon glucose starvation is disrupted in the absence of Get4–Get5 (Powis et al., 2013). Although Sgt2 is required for the enrichment of other GET proteins in GET bodies, its overexpression does not affect the number of GET bodies per cell (Fig. 2). This could suggest that multivalent interactions with the other components are limiting for GET body assembly, but the finding that the N-terminal region of Sgt2 is necessary for GET body formation also raises the possibility that dimerization of Sgt2 is important in this context.

The TPR region of Sgt2 is required for GET body formation (Chartron et al., 2011), but is dispensable for its function in canonical TA protein targeting in cells (Fig. 3), enabling the roles of Sgt2 as a targeting factor and in GET body formation to be discriminated. Consistent with the Sgt2 TPR region being a chaperone-interaction interface, analysis of enriched GET bodies highlighted numerous chaperones (Fig. 4; Table S2), including proteins of the Hsp70 family (Ssa1, Ssa2, Ssa4 and Sse2) (Figs 4 and 5). Also enriched with GET bodies were Sis1, a type II Hsp40 protein that functions as a co-chaperone for Ssa1, and Sti1, an Hsp90 co-chaperone that also interacts with Ssa proteins of the Hsp70 family and stimulates the ATPase activity of Ssa1 (Wegele et al., 2003). In response to acute glucose withdrawal, both Sis1 and Sti1 form foci independently of Sgt2 (Fig. 6), suggesting that they represent nucleating factors for GET bodies. Sis1 is present in various phase-separated granules (Klaips et al., 2020; Walters et al., 2015) and undergoes LLPS *in vitro* (Gu et al., 2020), a property that might rationalize its ability to initiate GET body formation. In addition, or alternatively, the presence of Sis1 in GET bodies might enable these assemblies to remain dynamic. Similar to Sgt2, Sti1 contains TPR regions; as TPR1 and TPR2B are binding sites for Hsp70 chaperones (Wegele et al., 2003), it is likely that they contribute to the function of Sti1 in GET body formation. An STI1 domain of Sti1 has been shown to promote LLPS (Zheng et al., 2021), and similar to the ability of Sis1 to phase separate, it is possible that this property contributes to its role as a nucleating factor for GET bodies and/or maintaining their dynamic state. The di- or oligo-merization of Hsp70-like chaperones is enhanced by ADP (Sarbeng et al., 2015; Thompson et al., 2012; Trcka et al., 2019), so the Hsp70 proteins in GET bodies formed during glucose starvation are likely to be in multimeric states. The network of interactions formed by the GET pathway components and the (co-)chaperones present in GET bodies may drive the assembly of these large structures.

Beyond the soluble GET pathway proteins and chaperones, our colocalization screen highlighted numerous additional proteins as GET body components (Table S3). Although it remains unknown whether these proteins associate due to their physical properties or for functional reasons, gene ontology searches highlighted terms linked to the detection of misfolded proteins, the cellular response to stress and metabolic processes. Although the former represent the chaperone-rich nature of GET bodies, the latter connection is in line with the induction of GET body formation by glucose withdrawal. Our results suggest that GET bodies might sequester components of pathways that are counter-indicated under the conditions of their formation, that is, the specific conditions of energy depletion that follow a phase of growth sustained by the fermentation of glucose. Consistent with this, we identified several enzymes involved in the anabolic reactions enabling secretion including Sec53, which facilitates N-linked glycosylation in the ER lumen (Feldman et al., 1987), Sam1, which catalyzes *S*-adenosylmethionine formation (Cherest and Surdin-Kerjan, 1978), Paa1, which contributes to formation of biogenic amines (Liu et al., 2005), and Hom2, which is required for amino acid synthesis (Mountain et al., 1991). Further linking GET bodies to regulation of the cellular metabolic status is the detection of enzymes linked to catabolic metabolism (e.g. Bud16, Fbp26 and Met3). Intriguingly, the portfolio of putative GET body components also includes several kinases and phosphatases, suggesting that regulation of the phosphoproteome might be associated with GET body formation or function.

A crucial property of GET bodies distinguishing them from protein aggregates is their rapid dissolution upon re-addition of glucose (Fig. 1). Sis1 and Sti1 likely contribute to maintaining the dynamic nature of GET bodies as in their absence, upon glucose withdrawal, Sgt2 forms distinct structures that probably represent aggregates (Fig. 6). Hsp70 chaperones in stress granules prevent the accumulation of misfolded proteins, thus inhibiting their transition to proteotoxic aggresomes (Mateju et al., 2017), and the Hsp70-family chaperones enriched in GET bodies might fulfill a similar function. Resolution of GET bodies is induced by addition of ethanol but not acetate (Fig. 7), highlighting that the reactions converting ethanol into acetate are an important trigger for GET body dissolution. Consistent with this, GET body dissolution is impaired by lack of Adh2, an alcohol dehydrogenase that primarily converts ethanol into acetaldehyde (Maestre et al., 2008). Addition of NADH, which is produced during the conversion of ethanol into acetate, to semi-permeabilized cells also leads to rapid dissolution of GET bodies (Fig. 7). Changes in the state of the NAD^+^/NADH redox potential is expected to affect the chaperone-holdase function of Get3 (Powis et al., 2013; Ulrich et al., 2022; Voth et al., 2014), which might partially explain the coupling of GET body dissolution to increases in cellular NADH. Exogenous ATP decreases stress granules numbers, but increases GET body numbers, and they appear smaller, which could reflect the activation of ATP-dependent chaperones, such as the disaggregase Hsp104 (Shorter and Southworth, 2019), Get3 or the Hsp70s, leading to fragmentation of GET bodies as a step towards their ultimate dissolution.

The ability of cells to respond to changing environmental conditions is crucial, and when metabolic re-programming is required, the cellular proteome is modulated to optimize efficient adaptation. The degradation of protein components of essential cellular processes in response to nutrient limitation would necessitate their re-expression to re-initiate growth, which might delay recovery. By contrast, sequestration of proteins in dynamic membraneless organelles allows utilization of pre-existing molecules. It is possible that the formation of GET bodies upon glucose withdrawal, on the one hand, extracts unnecessary, energy-consuming targeting factors and anabolic enzymes from the cytosol, and on the other hand, preserves a reservoir of such proteins to facilitate a rapid return to optimal growth conditions when possible.

Taken together, our results provide mechanistic insights into the regulation of a key membrane protein targeting pathway in response to acute glucose withdrawal, and contribute to the growing understanding of the roles that diverse membraneless organelles play in cellular adaptation to changing conditions.

## MATERIALS AND METHODS

### Plasmid construction

All plasmids used in this study are listed in Table S4. pRS415 *MET15*pr and pRS416 *MET15*pr were used as backbones for the plasmids generated in this study (Mumberg et al., 1994). The plasmids were generated using standard cloning techniques, including T4 ligation and Gibson assembly (Gibson et al., 2009). For T4 ligation, inserts were amplified with oligonucleotides listed in Table S5 and digested in parallel with the backbone with appropriate restriction enzymes at 37°C (Thermo Fisher Scientific). DNA fragments were separated in agarose gels and purified using High Pure PCR Product Purification Kit (Roche) according to the manufacturer's guidelines. The backbone and the insert were ligated using T4 ligase (Thermo Fisher Scientific) at 16°C for 2 h. *Escherichia coli* ElectroTenBlue (Agilent) were electroporated with the ligation mixture and plated onto LB agar plates supplemented with appropriate antibiotics. Independent colonies were used to amplify plasmids, and correct plasmids were confirmed by Sanger sequencing. For Gibson assembly, inserts with appropriate overhangs amplified by PCR and backbones were prepared as described above. Gibson assembly was carried out at 50°C for 1 h. The further procedure followed the workflow for T4 ligated plasmids.

### Yeast strain construction

Yeast strains used in this study derive from the genetic background BY4741 (Brachmann et al., 1998), unless otherwise indicated, and are listed in Table S6. Transformation of yeast with cassettes for genomic insertion via homologous recombination or plasmids was carried out using the lithium acetate-polyethylene glycol method (Gietz et al., 1992). Transformed strains using auxotrophic markers were spotted onto agar plates with synthetic dropout medium (Formedium) supplemented with 2 g/l glucose and incubated for 2 days at 30°C. When using antibiotic selection markers, cells were spotted onto YPD plates and incubated for 1 days at 30°C before being replica-plated onto YPD plates supplemented with appropriate antibiotics with 200 µg/ml G418 (Sigma-Aldrich), 100 µg/ml clonNAT (HKI Jena) or 37.5 µg/ml phleomycin (InvivoGen). Single colonies from genomically modified strains were re-streaked on appropriate plates and subsequently verified by genomic DNA extraction and PCR or protein extraction and western blotting. Antibiotic markers were amplified from a plasmid containing genes encoding resistances against clonNAT, G418 and phleomycin, derived from pCEV-G1 Km (Vickers et al., 2013). To tag proteins genomically with GFP, the encoding cassette was amplified from pKT128 (Sheff and Thorn, 2004) with an ∼40 basepair overhang on both ends to allow for homologous recombination.

Yeast strains for expression of proteins from plasmids were freshly transformed and selected on synthetic dropout medium (Formedium) prior to usage.

For the expression of N-terminally GFP tagged proteins, yeast strains from the N-SWAT library (Weill et al., 2018) were used. Yeast strains expressing mNG-tagged proteins under the control of their native promoter were obtained from the C-SWAT library (Meurer et al., 2018).

### Yeast growth and medium exchanges

All experiments were carried out with yeast cells in the logarithmic growth phase if not otherwise indicated. Strains carrying plasmids, used for microscopy, or to be starved for glucose were cultured in synthetic dropout medium (Formedium) with appropriate auxotrophic markers. Other experiments were performed with yeast growing in YPD medium. To starve cells of glucose, cells from logarithmic cultures were harvested by centrifugation (3000 ***g*** for 1 min) and re-suspended in SC medium without glucose. This step was repeated twice, and the cells were then incubated for the indicated times in low fluorescence SC medium (Formedium, lacking folic acid and riboflavin) without glucose at 30°C with agitation. The recovery of glucose-starved cells was induced by addition of glucose to a final concentration of 2% or lower as indicated. Also, cells were grown in medium containing different carbon sources. Cells were centrifuged at 3000 ***g*** and re-suspended in SC medium containing either acetate (2% w/v, pH adjusted) or ethanol (EtOH, v/v, 2%). At these concentrations, acetate (Paiva et al., 2004) and ethanol (Kotyk and Alonso, 1985) will enter the cells.

### Growth analysis of yeast strains

The growth of strains was monitored in liquid SC medium (Formedium) in 100 ml shaking flasks. Every hour, a sample was taken and the absorption at 600 nm (OD_600_) was measured. For each culture, the growth constant and the doubling time was calculated. The datapoints were fitted to an exponential regression model *y*=a×*e*^(*k*×*x*)^, with *k* being the growth constant.

### Microscopy

Yeast strains with and without plasmids were cultured in synthetic dropout medium (Formedium) with the appropriate markers until they reached logarithmic growth phase before imaging. 15 µl of cultures were mixed with 35 µl of low-fluorescence minimal medium (Formedium) in 384-well glass-bottom plates (Azenta). Images for colocalization assays were acquired with microscopy slides and cover slips. Microscopy images were taken as described previously (Farkas et al., 2022). In brief, a Nikon Ti2 2-E inverted microscope equipped with a computer-controlled stage, a Lumencor Spectra X light source and a pco.edge 5.5 M-AIR-CL-PCO sCMOS camera (2560×2156 pixels) using NIS-Elements software (Nikon) was employed to acquire images at room temperature. The Perfect Focus System (Nikon) detected the focal plane. A CFI Plan Apo Lambda 100×/1.45 oil objective together with appropriate settings for GFP (excitation 470 nm; emission, 520/35 nm) and mTagBFP2 (excitation 395 nm; emission, 433/24 nm) was used. Image analysis, cropping, brightness adjustment, conversion from 16-bit into RGB and figure preparation was carried out using Fiji software (Schindelin et al., 2012).

For imaging in the TA targeting assay, stationary-phase cells were diluted in 384-well glass-bottom plates (Azenta) with low-fluorescence medium (Formedium) supplemented with 2% glucose and the appropriate dropout components, and incubated for 4 h at 30°C. Cells were then imaged as described previously (Rivera-Monroy et al., 2016). In brief, an Imaging Machine 03-dual (Acquifer) widefield high-content screening microscope, equipped with LED arrays for brightfield and fluorescence excitation, movable optics, and stationary plate holder was employed. Images were acquired with an sCMOS camera (2048×2048 pixels), a 40× CFI Super Plan Fluor ELWD N.A. 0.60 (Nikon), and with 470 nm filter cubes (excitation 469/35 nm; emission, 525/39 nm, dichroic 497 nm) or without filter cube for brightfield images in three *z*-slices (dz=1 µm). The focal plane was automatically detected in the brightfield channel using a yeast autofocus algorithm.

### Evaluation of microscopy images

Image analysis, cropping, brightness adjustment, conversion from 16-bit into RGB and figure preparation was carried out using Fiji software (Schindelin et al., 2012). To calculate the percentage of yeast cells with GET foci, at least 100 representative yeast cells were inspected manually. The number of cells with GET foci was divided by the total number of living cells surveyed. The numbers of cells with one focus and with multiple foci were added and divided by the total number of living cells surveyed.

For assessing whether proteins colocalize with GET foci marker proteins, the proteins were tagged with proteins fluorescing in different channels. The images were inspected manually to allow for detection of dead cells. At least 100 foci were counted for both proteins and the percentages of overlapping foci and foci containing only one protein were calculated. The results are depicted in Euler diagrams created with an online tool (https://eulerr.co/; https://CRAN.R-project.org/package=eulerr), in which the areas of ellipses are proportional to the number of foci.

All microscopy images presented are representative of three biological replicates with >100 cells imaged in seven *z*-planes for each replicate.

### Cell lysis for protein extraction and western blotting

Preparation of samples for analysis of proteins for western blotting was adapted from a previous published protocol (Farkas et al., 2022; Kushnirov, 2000). In brief, cells were cultured in synthetic dropout medium until they reached logarithmic growth phase, pelleted and re-suspended in 1 ml of 250 mM NaOH. Cells were incubated on ice for 10 min, pelleted and re-suspended in NuPAGE LDS sample buffer (Thermo Fisher Scientific) (OD_600_ of the cells in NaOH×100=µl of sample buffer used to resuspend the cell pellet). Samples were boiled at 70°C for 5 min, centrifuged at 16,000 ***g*** for 30 s and stored at −20°C. 10 µl were used for analysis by western blotting. Proteins were resolved according to their size in Bis-Tris-gels and transferred onto PVDF membranes. The membranes were blocked with 5% milk in Tris-buffered saline (TBS). Primary antibodies (Table S7) were added in TBS containing 0.1% Tween 20. Afterwards, membranes were washed with TBS containing 0.1% Tween 20 and secondary antibodies (Table S7) were applied in TBS containing 0.1% Tween 20 and 0.01% sodium dodecyl sulfate. Membranes were scanned in a LI-COR Odyssey scanner and images were quantified in Image Studio 5.2.5 (LI-COR). The antibodies used in this study were validated using knockout yeast strains (for Get3 and Pgk1) or stains expressing untagged proteins (anti-GFP, anti-HA and anti-mNeonGreen antibodies). All western blots presented were performed in triplicate and representative data are shown in the figures. Raw images of all western blots performed in this study are presented in Fig. S6.

### Total RNA extraction from yeast

Cells were grown in SC medium with the appropriate dropout in logarithmic phase before harvesting (3000 ***g*** for 10 min). The cell pellet was flash frozen and 0.2 ml GTC mix (2 M guanidine thiocyanate, 25 mM Tris-HCl pH 8.0, 5 mM EDTA pH 8.0, 1% N-lauroylsarcosine, 150 mM β-mercaptoethanol), 0.2 ml phenol and 0.6 ml of glass beads were added. Cells were disrupted by vortexing four times for 1 min with intervals of 1 min on ice. Then, 3 ml GTC mix and 3 ml acidic phenol were added and the samples were incubated for 5 min at 65°C and then on ice for 5 min. 1.6 ml sodium acetate mix (100 mM sodium acetate pH 5.2, 1 mM EDTA pH 8.0, 10 mM Tris/HCl pH 8.0) and 3 ml chloroform were added and vortexed before centrifugation at 4000 ***g***. The upper aqueous phase was removed, mixed with 5 ml PCI (phenol:chloroform:isoamyl alcohol in a 25:24:1 ratio) and again centrifuged at 4000 ***g*** for 20 min. The aqueous phase was removed and mixed with 4.5 ml chloroform, then centrifuged at 4000 ***g*** for 20 min. The aqueous phase was mixed with 100% ethanol and stored overnight at −20°C to allow precipitation. Samples were centrifuged at 4000 ***g*** for 30 min at 4°C and the supernatant discarded. The pellet was washed with cold 70% ethanol, then air-dried and re-suspended in 50 µl water. The concentration of RNA was determined with a NanoDrop (Thermo Fisher Scientific).

### Quantitative PCR

Analysis of the expression levels of the endogenous *GET4* and *GET5* mRNAs in a wild-type strain compared to a strain ectopically overexpressing both mRNAs was performed with reverse transcriptase quantitative PCR (RT-qPCR). For reverse transcription, 2 µg of purified total RNA were mixed with an anchored oligo d(T) primer, Superscript III reverse transcriptase (200 U, Thermo Fisher Scientific) and 1 mM dNTPs in 1× first-strand buffer (Thermo Fisher Scientific) supplemented with 5 mM dithiothreitol (DTT). The reaction was carried out at 50°C for 1 h followed by heat-inactivation of the enzyme at 70°C for 15 min. The cDNA was purified using the QIAquick PCR purification kit (Qiagen) according to the manufacturer's instructions. The concentration of the cDNA was measured with the NanoDrop and equal amounts of cDNA were used for qPCR. Primers for the qPCR were designed with the same melting temperature and to generate equally long amplicons of 150 bp. Amplification of a single amplicon by the designed primers was tested with a melting curve analysis. The efficiency and linearity of the amplification was determined and primers with an efficiency below 90% were excluded. The qPCR reactions were performed in a LightCycler 480 II (Roche). In the reaction, 1× SYBR^®^ Green Jumpstart Taq Readymix (Sigma) was mixed with the primers and 2 µl of equilibrated cDNA. The cycling conditions were 95°C for 5 min, 40 cycles of 95°C for 30 s, 55°C for 30 s, 72°C for 30 s, followed by 95°C for 1 min, 55°C for 30 s and 95°C for 30 s. Each reaction was performed in technical triplicates. The values for *GET4* and *GET5* mRNA were normalized to the values for the housekeeping gene *TAF10*. The fold-change was calculated with the comparative Ct method with the arithmetic formula 2^−ΔΔCt^.

### TA protein targeting assay

A script written in KNIME (https://www.knime.com) that identifies cells and quantifies the distribution of fluorescence within them automatically was used (McDowell et al., 2020). In brief, an Fiji (Schindelin et al., 2012) macro in KNIME identified cells by calculating the difference of two brightfield images (±1 µm from focal plane). The background was identified as areas that contained no irregularities and had little variance. Cells that overlapped with calculated background area were excluded from further evaluation. Moreover, cells were filtered for circularity, variance in the brightfield signal within the cell, size (i.e. cells that were too big or small), and were excluded if they did not fit the borders. Also, the range of brightfield signal values within cells were filtered to excluded cells that contained irregularities or were stacked on each other from analysis. The background of the fluorescent channel was measured in the areas defined as background with the brightfield images and then subtracted from the fluorescent channel image. The pixel distribution in the fluorescent channel was calculated for each cell identified in the brightfield image. To remove dead cells from the analysis, cells with too little variance within them were excluded as well as cells with too high signal intensity. Cells with too low signal intensity were also removed, thus excluding cells not properly expressing GFP–Sed5. The ratio of the average fluorescence signal from the cell and the maximum puncta signal was calculated and averaged for all cells in the image. Ten images per strain with at least 300 cells were used for quantification.

### GET body enrichment

The procedure for GET body enrichment was adapted from a previously published method for enrichment of stress granule cores (Wheeler et al., 2017). Cells were cultured in synthetic complete dropout medium (1.9 g/l yeast nitrogen base without amino acids, 5 g/l ammonium sulfate, 2% D-glucose, 0.79 g/l complete amino acid mixture; Formedium) expressing either Sgt2–GFP or Sgt2ΔTPR–GFP from a plasmid, driven by the *MET25pr*. Cells were three times washed and then re-suspended in glucose-free synthetic complete medium and incubated for 1 h at 30°C. Equal amounts of cells were harvested by centrifugation (3000 ***g*** for 4 min), snap-frozen and stored at −80°C. Pellets were re-suspended in 1 ml of lysis buffer [50 mM Tris-HCl pH 7.4, 100 mM potassium acetate, 2 mM magnesium acetate, 0.5 mM DTT, 0.5% IGEPAL (Sigma-Aldrich), 1 tablet/25 ml cOmplete EDTA-free protease inhibitor cocktail (Roche)] and lysed with glass beads by vortexing three times for 3 min at 4°C with 2 min pauses on ice in-between. The lysate was centrifuged twice at 500 ***g*** and 4°C to remove cell debris. The cleared lysate was centrifuged at 4000 ***g*** and 4°C to pellet the GET bodies. The pellet was re-suspended in 100 µl of lysis buffer and centrifuged again at 2000 ***g*** and 4°C to remove remaining cell debris. The isolated GET bodies were imaged immediately. 40 µl of ChromoTek GFP-Trap^®^ magnetic agarose beads (Proteintech) per sample were added to the GET body-enriched fraction and incubated at 4°C for 30 min with shaking. GET bodies captured on the beads were imaged. The beads were washed three times with lysis buffer without protease inhibitor, re-suspended in about 25 µl of lysis buffer without protease inhibitor containing 5 µg of TEV protease (Schwappach laboratory), and incubated for 1 h at room temperature. Input and eluate were mixed with appropriate amounts of NuPAGE LDS sample buffer (Thermo Fisher Scientific), boiled for 5 min at 70°C and further used for western blotting and mass spectrometric analysis.

### Mass spectrometry analysis of enriched GET bodies

The eluates from the TEV protease-treated samples from the GET body enrichment were incubated at 70°C for 5 min and then resolved on NuPAGE 4-12% Bis-Tris gels. The lanes were excised and divided into seven gel pieces per lane. The pieces were washed with acetonitrile and then incubated with 10 mM DTT to reduce disulfide bridges. Subsequently, reduced cysteine residues were alkylated by incubation with 55 mM iodoacetamide. Proteins were digested in-gel by adding trypsin (Sigma-Aldrich) and incubation overnight at 37°C. The peptides were extracted using acetonitrile and 5% formic acid and dried completely. Then, they were resolved in sample buffer (2% acetonitrile, 0.1% formic acid) and injected into a nano-LC system.

The via MaxQuant (Cox and Mann, 2008) identified yeast peptides were filtered to exclude contaminants, reverse peptides, decoy peptides, and peptides not identified in all three replicates. The intensity values were transformed into log_2_ numbers and missing values were imputed with numbers from a normal distribution employing the software Perseus (Tyanova et al., 2016). Using Welch's *t*-test, the significance of the difference between the samples was calculated. In the analysis, proteins were highlighted that were significantly (*P*<0.05) enriched more than four-fold [>log (2)] with Sgt2 compared to Sgt2ΔTPR.

### High-throughput microscopy-based screening

To identify proteins colocalizing with GET bodies upon acute glucose starvation, the C-SWAT library was used (Meurer et al., 2018). In each of the 5661 strains of this library, a specific protein is tagged at the C-terminus with mNG and expressed under the control of their native promoter. To generate a C-SWAT-derived library expressing mTagBFP2–Get3 from a plasmid, the synthetic genetic array (SGA) method was used (Cohen and Schuldiner, 2011; Yan Tong and Boone, 2006). The C-SWAT library was mated with a query strain containing the plasmid p415 mTagBFP2–Get3 using a Singer robot, and diploid cells were selected using the appropriate markers. The cells were cultured in synthetic dropout medium in 384-well plates overnight. 1 µl of stationary culture was mixed with 30 µl fresh low fluorescence medium (Formedium) lacking methionine, as the diploid strains are methionine-prototroph and the *MET25* promoter present in the plasmid is more active under low methionine concentrations. Cells were incubated in 384-well glass-bottom plates (Azenta) at 30°C for 4 h. The medium was slowly removed with a liquid handler (Agilent) and the cells were washed with glucose-free low fluorescence media. The medium was removed again and 50 µl of low fluorescence medium without glucose but containing 2% 2-deoxy-glucose were added. The cells were incubated at 30°C for 1 h and then imaged.

### Degradation of essential proteins

To deplete essential proteins, the auxin inducible degron (AID) system was employed as described previously (Morawska and Ulrich, 2013; Nishimura et al., 2009). In brief, a yeast strain expressing the Tir1 protein from *Oryza sativa* under the control of the *ADH1* promoter was used as parental strain (Nishimura et al., 2009) and the essential protein was genomically tagged with the AID* tag, which consists of amino acids 71–114 of IAA17 (Morawska and Ulrich, 2013). The encoding was amplified from P03F06 with ∼40 basepair overhang on both ends to allow for homologous recombination. To degrade AID*-tagged proteins, indole-3-acetic acid (IAA) solved in ethanol was added to the cultures to a final concentration of 500 µM 1 h before the experiment.

### Cell permeabilization and exogenous addition of metabolites

Cells were treated with 0.1% digitonin for 15 min to semi-permeabilize the plasma membrane (Berłowska et al., 2006). NADH to a final concentration of 7.5 mM or ATP to a final concentration of 500 µM in SC medium were added, and cells were incubated for the indicated times.

### Data quantification and statistical analyses

Unless otherwise stated, all bar charts represent the average of three biological replicates with individual data points shown as gray dots. Error bars indicate standard deviation of the mean. Colocalization is shown as Euler diagrams, where the size of the circles represents the proportion of the total foci counted. The *P*-values calculated using Welch's *t*-test are shown with numbers and represented as follows: n.s., non-significant; *P*>0.05; *P<0.05; **P<0.01 and ***P<0.005.

## Supplementary Material

10.1242/joces.263616_sup1 Supplementary information

Table S1. Co-localization of GET bodies with foci containing different proteins

Table S2. Inventory of proteins co-enriched with GET bodies 4 h after glucose withdrawal.

Table S3. Proteins co-localizing with GET bodies by high-throughput microscopy screening.

Table S4. Plasmids used and generated in this study.

Table S5. Oligonucleotides used in this study.

Table S6. Yeast strains used in this study.

Table S7. Antibodies used in this study
