# Supplementary material for: The formation of chaperone-rich GET bodies depends on the tetratricopeptide repeat region of Sgt2 and is reversed by NADH
Source: J Cell Sci. 2025 Mar 20;138(6):jcs263616. doi: 10.1242/jcs.263616 (PMC11959614; doi:10.1242/jcs.263616)
Supplement: Supplementary information [file joces-138-263616-s1.pdf]

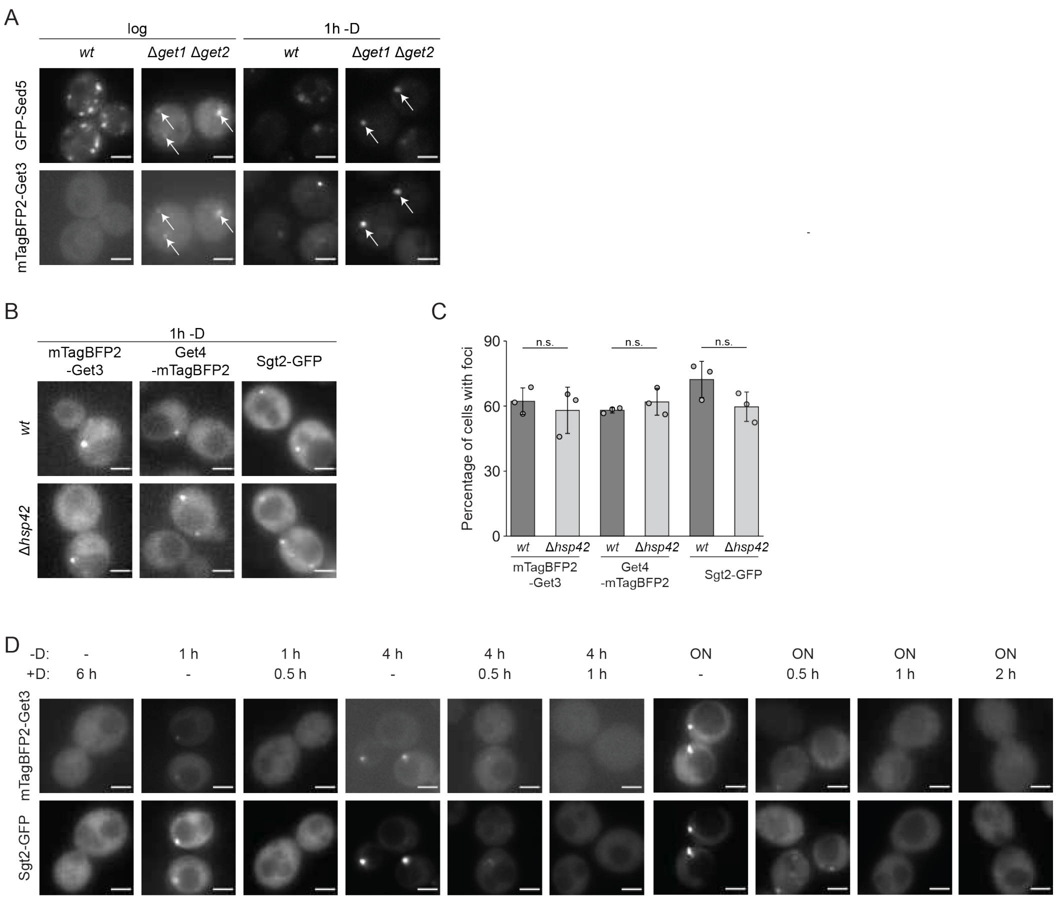

Fig. S1. Glucose starvation-induced GET bodies are dynamic structures distinct from those formed upon impaired GET pathway targeting or those requiring Hsp42 for assembly. (A) Fluorescence microscopy of *wt* or Δ*get1*Δ*get2* strains expressing GFP-Sed5 and mTagBFP2-Get3 in log phase or after 1 h of glucose removal (1h -D). Arrows indicate co-localization. **(B)** Fluorescence microscopy of strains expressing mTagBFP2-Get3, Get4-mTagBFP2 or Sgt2-GFP in a *wt* or Δ*hsp42* background after 1 h glucose starvation. **(C)** Percentages of cells in (D) with foci. **(D)** Fluorescence microscopy of cells expressing Sgt2-mNG and mTagBFP2-tagged Get3 from the *MET25* promoter in log phase or after the indicated times in media lacking glucose (-D). Glucose was re-added for the indicated times (+D). All scale bars: 2 µm. n=3 biological replicates. Error bars = mean±s.d., p-values calculated using Welch's t-test.

Journal of Cell Science • Supplementary information

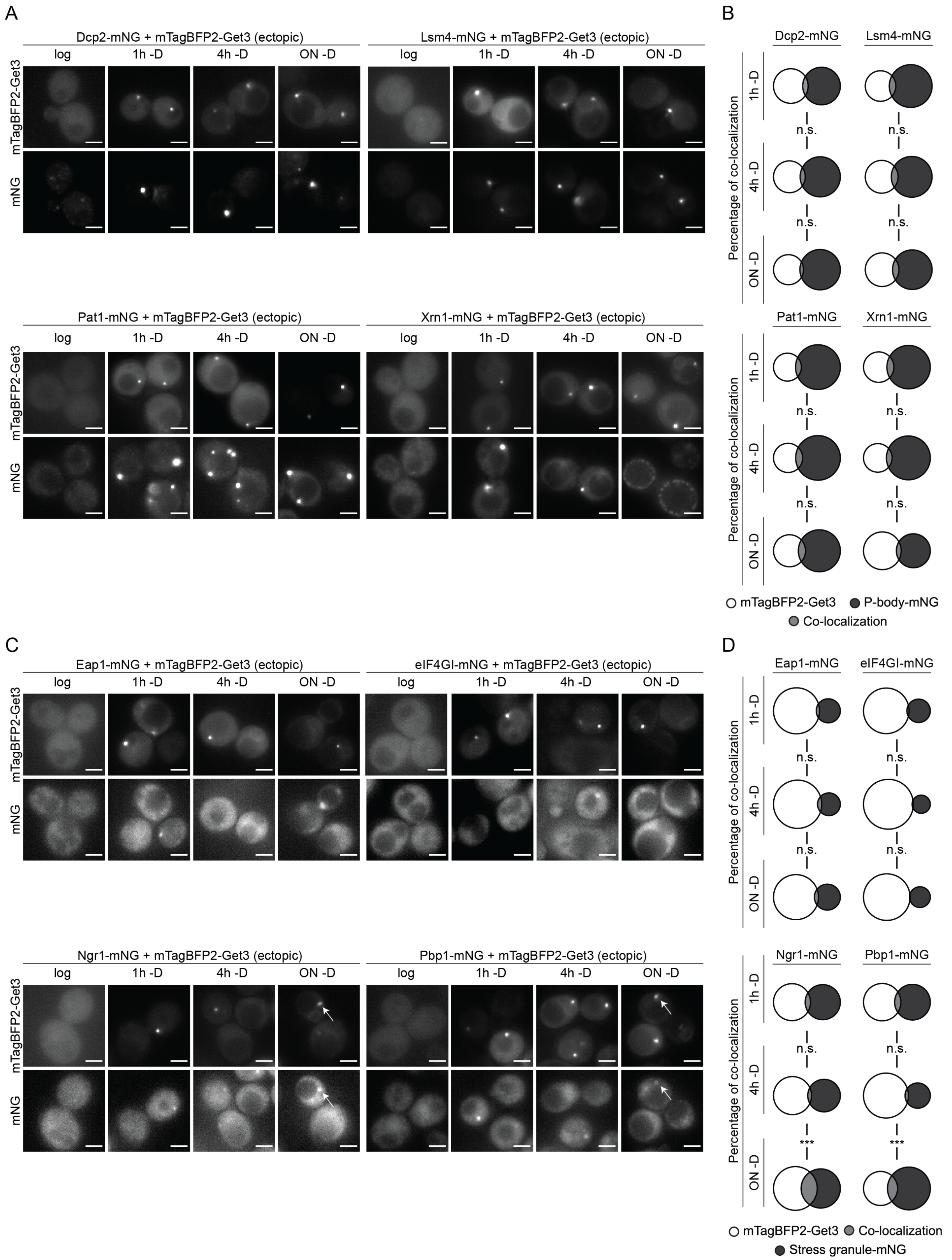

Fig. S2. GET bodies are distinct from P-bodies and stress granules, but share components with stress granules after prolonged glucose withdrawal. (A-D) Fluorescence microscopy of yeast expressing mNG-tagged P-body (A) or stress granule (C) marker proteins and mTagBFP2-Get3 in log phase and after 1 h, 4 h and overnight (ON) glucose starvation (-D). Scale bars: 2 µm. Percentages of co-localization between mTagBFP2-Get3-positive foci and foci containing a P-body (B) or stress granule (D) marker. Sizes of circles represent the proportions of the total foci counted. n=3 biological replicates.

Journal of Cell Science • Supplementary information

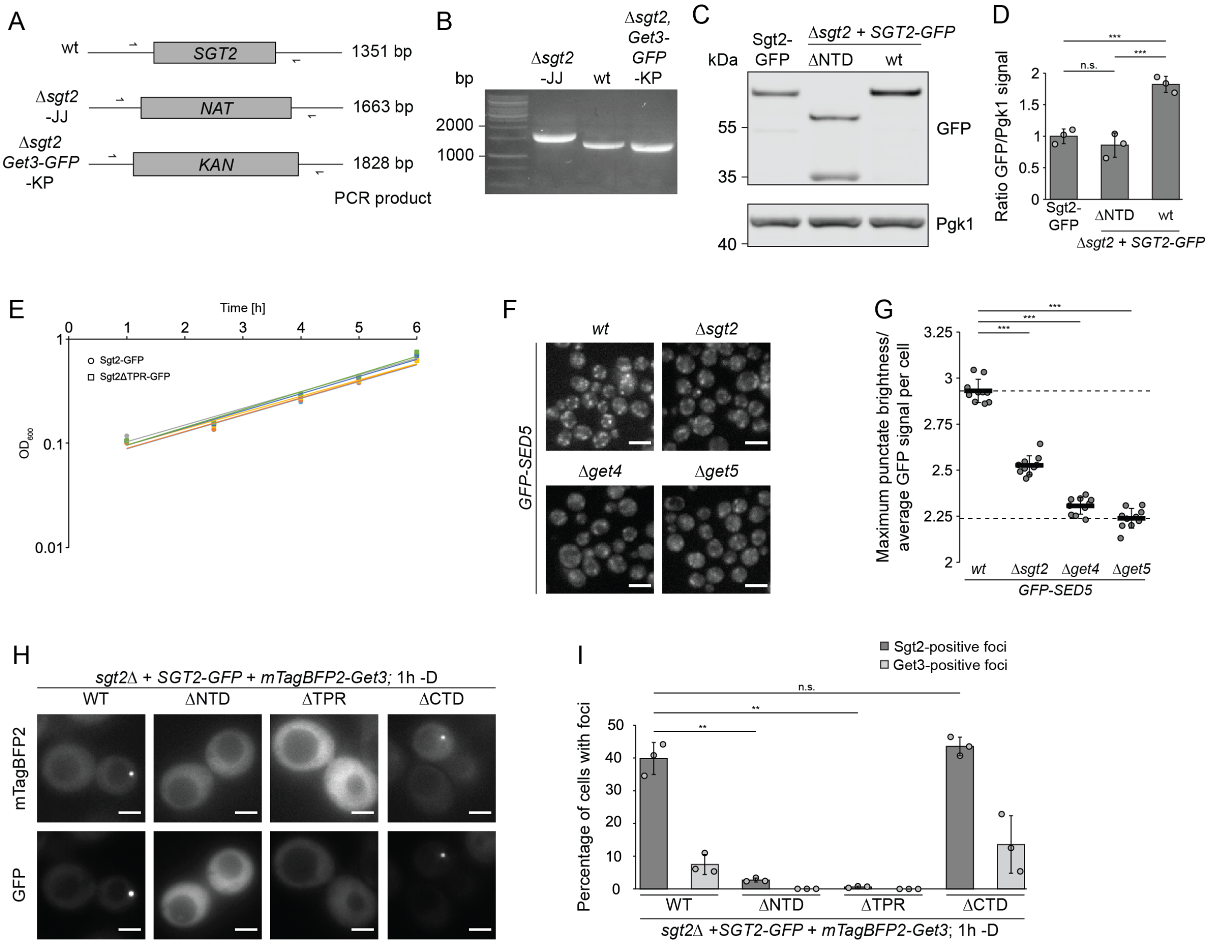

Fig. S3. Requirement of Sgt2 regions for TA protein targeting and GET body formation. (A) Schemes of the wild-type (wt) *SGT2* locus and the replacement of the *SGT2* coding sequence by *NAT* or *KAN* cassettes. Positions of PCR primers in (B) are shown. JJ – parental Δ*sgt2* strain used in this study; KP – the Δ*sgt2, Get3-GFP* strain used in (Powis et al., 2013). **(B)** PCR products amplified from genomic DNA extracted from the strains in (A). **(C)** Immunoblot of proteins from wt cells expressing Sgt2-GFP and Δ*sgt2* expressing Sgt2-GFP or Sgt2ΔNTD from the *MET25* promoter. **(D)** Quantification of SGt2-GFP (C), normalized to the signal of Pgk1. **(E)** Growth analysis of strains expressing Sgt2-GFP or Sgt2ΔTPR-GFP endogenously. **(F)** Fluorescence microscopy images of a strain expressing GFP-Sed5 endogenously from the *NOP1* promoter in wt (Δ*met15*) and Δ*sgt2*, Δ*get4*, Δ*get5* and quantification of the GFP-Sed5 distribution shown in images. **(G)** Quantification of (F). Upper and lower dashed lines represent the ratio between maximum punctate brightness and the average GFP signal per cell in the wt strain and the Δ*get5* strain, respectively. **(H)** Fluorescence microscopy of Δ*sgt2* expressing Sgt2-GFP or the truncated variants as well as mTagBFP2-Get3 in log phase or after 1 h without glucose (-D). **(G)** Quantification of the percentages of cells with foci in (H). Scale bars: 2 μm. n=3 (B-E, H-I) or n=10 (F-G) biological replicates. Error bars = mean±s.d., p-values calculated using Welch's t-test.

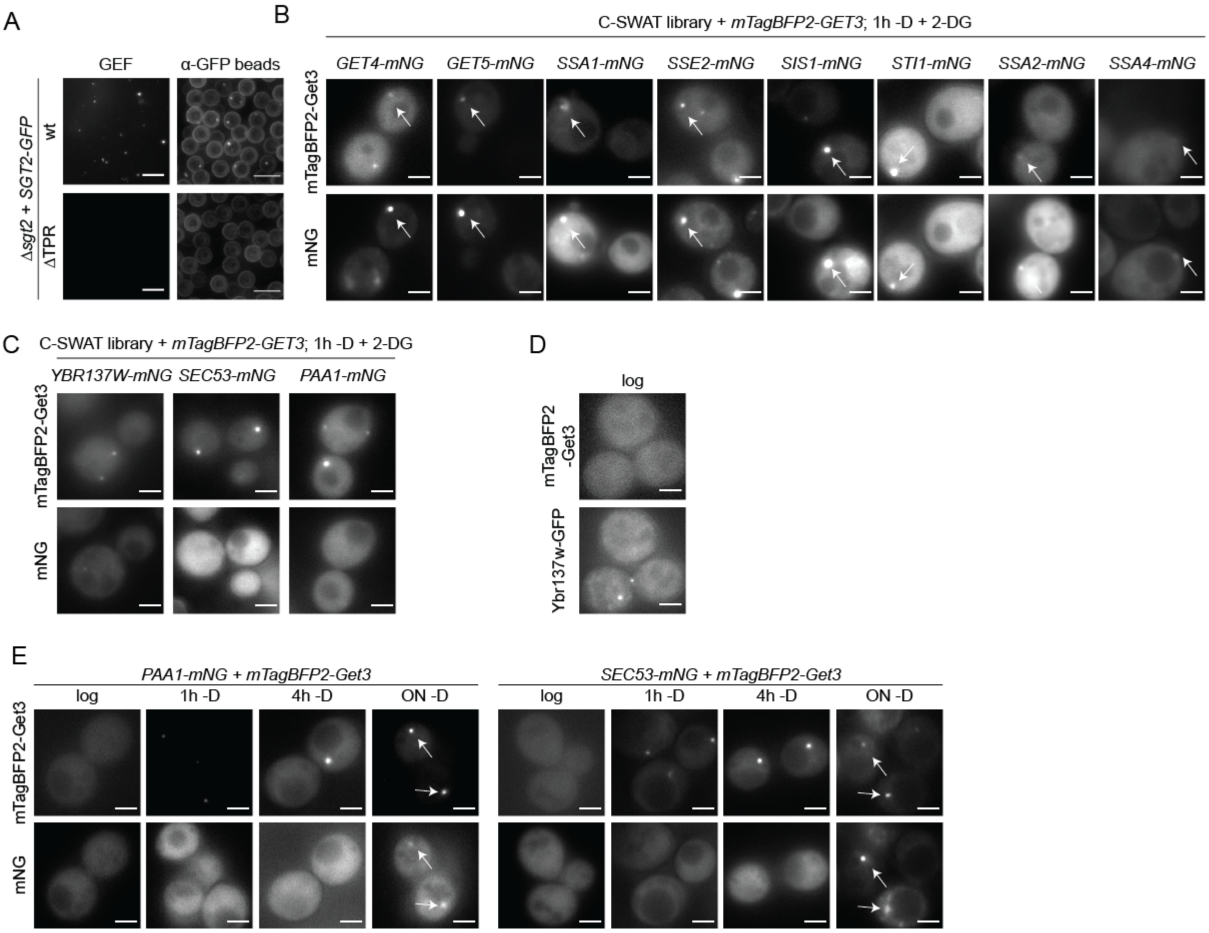

Fig. S4. Co-localization of putative GET body components with Get3-containing foci. (A) Fluorescence microscopy of the GET body enriched fraction (GEF) from Δ*sgt2* strain expressing Sgt2-TEV-GFP or Sgt2ΔTPR-TEV-GFP and captured GET bodies on α-GFP bead matrix. Scale bars: 2 µm for GEF, 5 µm for beads. **(B, C)** Fluorescence microscopy of strains expressing mNG-tagged proteins and mTagBFP2-Get3 from the *MET25* promoter after 1 h without glucose and 2% 2-deoxyglucose (2-DG). **(D)** Fluorescence microscopy of *wt* expressing Ybr137w-GFP and mTagBFP2-Get3 from the *MET25* promoter in log phase. **(E)** Fluorescence microscopy strains expressing Paa1-mNG or Sec53-mNG and mTagBFP2-Get3 from the *MET25* promoter in log phase and after 1 h, 4 h and overnight (ON) without glucose (-D). Scale bars: 2 µm. n=3 biological replicates.

Journal of Cell Science • Supplementary information

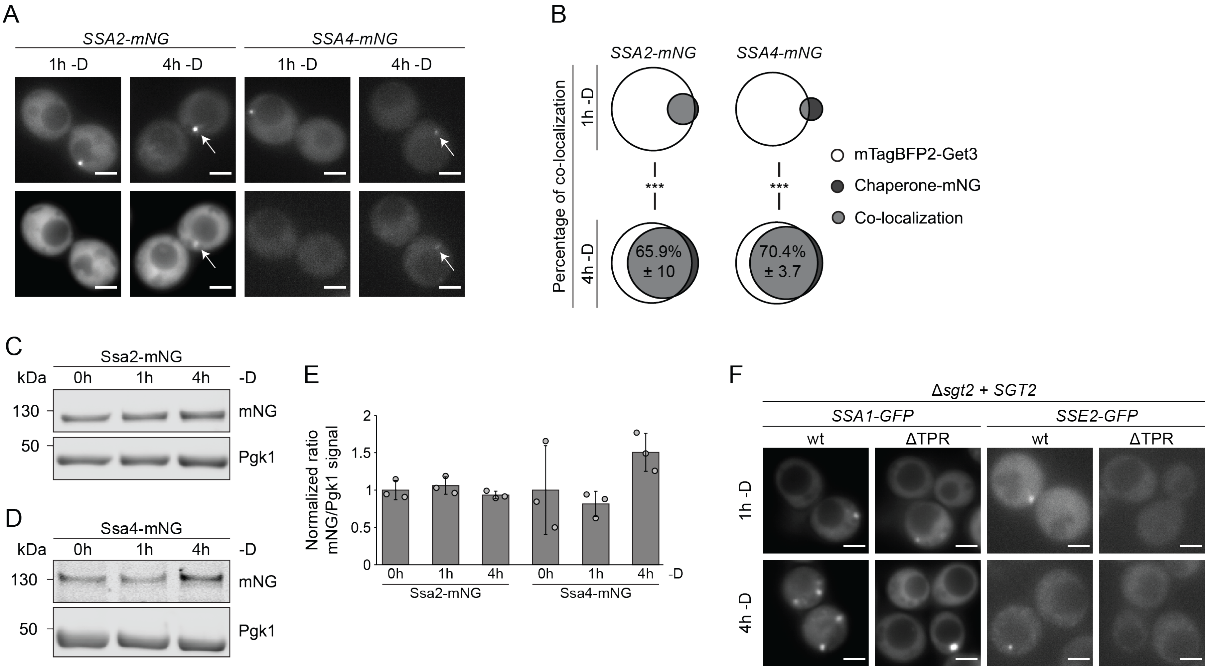

Fig. S5. Association of Hsp70 -like chaperones with GET bodies. (A-B) Proportions of co-localization between mTagBFP2-Get3-positive foci and Ssa2-mNG or Ssa4-mNG-positive foci. Sizes of circles represent the proportions of the total foci counted. **(C-E)** Immunoblots of proteins from cells expressing Ssa2-mNG (C) or Ssa4-mNG (D) and quantification (E) of mNG in (C-D), normalized to Pgk1. **(F)** Fluorescence microscopy of Δ*sgt2* strains expressing Ssa1-GFP or Ssa4-GFP and Sgt2 or Sgt2ΔTPR from the *MET25* promoter after 1 h and 4 h without glucose (-D). n=3 biological replicates. Error bars = mean ±s.d. and p-values were calculated using Welch's t-test.

Journal of Cell Science • Supplementary information

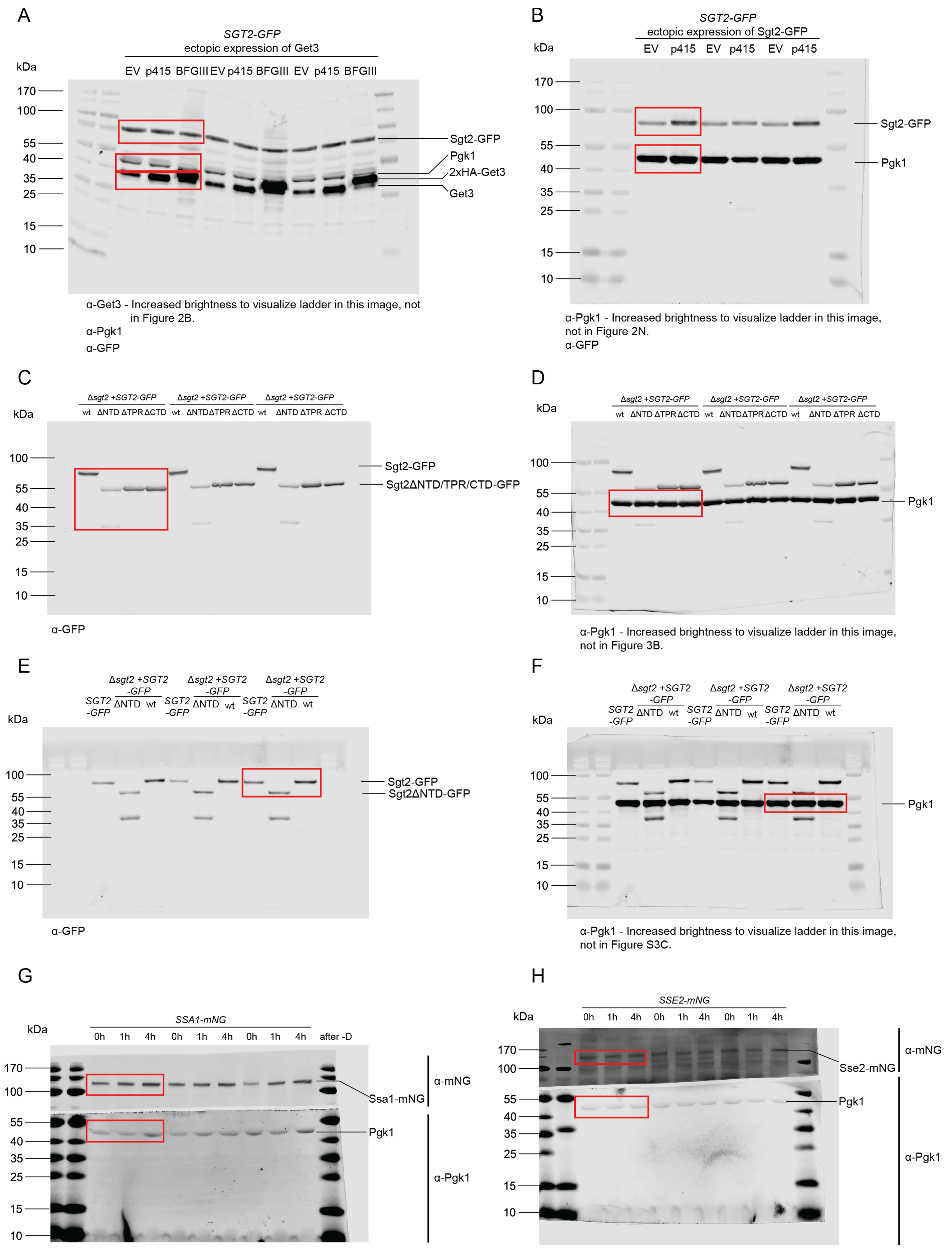

Journal of Cell Science • Supplementary information

I

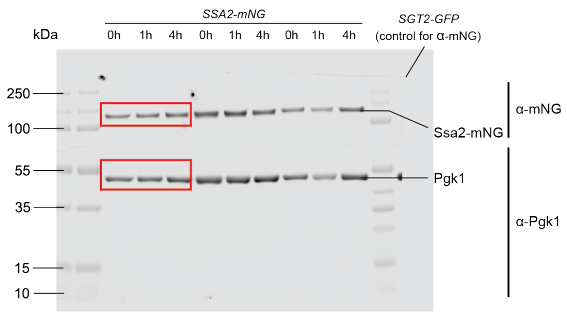

J

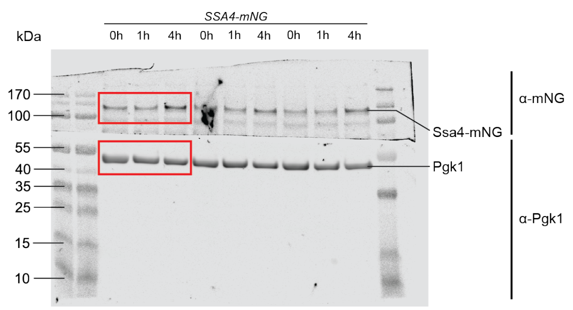

K

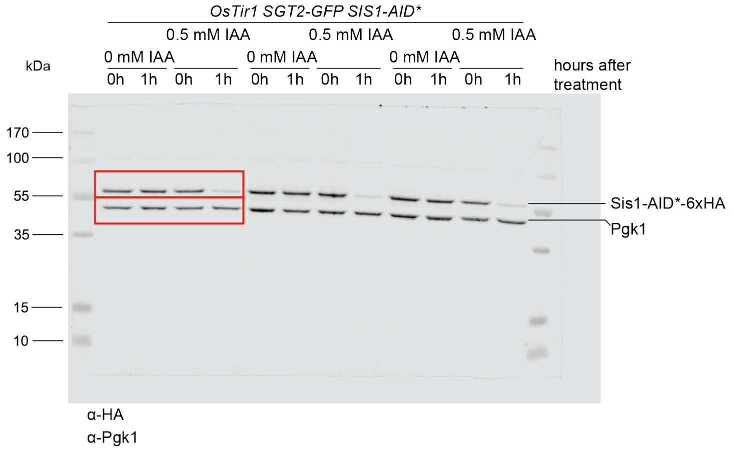

Fig. S6. Unprocessed images of western blots presented in this study. (A) Unprocessed images corresponding to Figure 2B. **(B)** Unprocessed images corresponding to Figure 2N. **(C-D)** Unprocessed images corresponding to Figure 3B. **(E-F)** Unprocessed images corresponding to Supplementary Figure S3C. **(G)** Unprocessed image corresponding to Figure 5C. **(H)** Unprocessed images corresponding to Figure 5D. **(I)** Unprocessed images corresponding to Supplementary Figure S5C. **(J)** Unprocessed images corresponding to Supplementary Figure S5D. **(K)** Unprocessed images corresponding to Figure 6C.

Journal of Cell Science • Supplementary information

Table S1. Co-localization of GET bodies with foci containing different proteins

Available for download at
https://journals.biologists.com/jcs/article-lookup/doi/10.1242/jcs.263616#supplementary-data

Table S2. Inventory of proteins co-enriched with GET bodies 4 h after glucose withdrawal.

Available for download at
https://journals.biologists.com/jcs/article-lookup/doi/10.1242/jcs.263616#supplementary-data

Table S3. Proteins co-localizing with GET bodies by high-throughput microscopy screening.

Available for download at
https://journals.biologists.com/jcs/article-lookup/doi/10.1242/jcs.263616#supplementary-data

Table S4. Plasmids used and generated in this study.

Available for download at
https://journals.biologists.com/jcs/article-lookup/doi/10.1242/jcs.263616#supplementary-data

Table S5. Oligonucleotides used in this study.

Available for download at
https://journals.biologists.com/jcs/article-lookup/doi/10.1242/jcs.263616#supplementary-data

Table S6. Yeast strains used in this study.

Available for download at
https://journals.biologists.com/jcs/article-lookup/doi/10.1242/jcs.263616#supplementary-data

Table S7. Antibodies used in this study

Available for download at
https://journals.biologists.com/jcs/article-lookup/doi/10.1242/jcs.263616#supplementary-data

SUPPLEMENTARY REFERENCES

Powis, K., Schrul, B., Tienson, H., Gostimskaya, I., Breker, M., High, S., Schuldiner, M., Jakob, U. and Schwappach, B. (2013). Get3 is a holdase chaperone and moves to deposition sites for aggregated proteins when membrane targeting is blocked. *J. Cell Sci.* **126**, 473–483.
